# Executive function abilities in cognitively healthy young and older adults—A cross-sectional study

**DOI:** 10.3389/fnagi.2023.976915

**Published:** 2023-02-08

**Authors:** Mojitola I. Idowu, Andre J. Szameitat

**Affiliations:** Department of Life Sciences, Division of Psychology, Centre for Cognitive and Clinical Neuroscience (CCN), College of Health, Medicine and Life Sciences, Brunel University London, Uxbridge, United Kingdom

**Keywords:** cognitive decline, cognitive aging, cognitive abilities, executive functions, dual-tasking, inhibition, shifting, working memory updating

## Abstract

A prominent feature of cognitive aging is the decline of executive function (EF) abilities. Numerous studies have reported that older adults perform poorer than younger adults in such tasks. In this cross-sectional study, the effect of age on four EFs, inhibition, shifting, updating, and dual-tasking, was examined in 26 young adults (mean 21.18  years) and 25 older adults (mean 71.56  years) with the utilization of a pair of tasks for each EF. The tasks employed for DT were the Psychological Refractory Period paradigm (PRP) and a modified test for everyday attention, for inhibition the Stroop and Hayling sentence completion test (HSCT), for shifting a task switching paradigm and the trail making test (TMT), and for updating the backward digit span (BDS) task and a n-back paradigm. As all participants performed all tasks, a further aim was to compare the size of the age-related cognitive decline among the four EFs. Age-related decline was observed in all four EFs in one or both of the tasks employed. The results revealed significantly poorer performance in the older adults in the response times (RTs) of the PRP effect, interference score of the Stroop, RT inhibition costs of the HSCT, RT and error-rate shifting costs of the task switching paradigm, and the error-rate updating costs of the n-back paradigm. A comparison between the rates of decline revealed numerical and statistically significant differences between the four EFs, with inhibition showing the greatest decline, followed by shifting, updating, and dual-tasking. Thus, we conclude that with age, these four EFs decline at different rates.

## Introduction

Cognitive decline is a well-known aspect of the healthy aging process, which adversely affects cognitive abilities, i.e., the mental capacity and skills required to perform tasks, of which executive functions (EFs) are considered subsets (Cabeza et al., 2004; Craik and Salthouse, 2008; Salthouse, 2009, 2012). Many factors, including diet, well-being, educational attainment, and physical health contribute to the neural, psychophysiological, and anatomical process of aging and, thus, influence the amount of age-associated decline observed in older adults (Haier et al., 2003; Friedman et al., 2008; MacPherson et al., 2019). However, it is understood that individuals with advanced education, better occupations, and/or better lifestyles tend to be able to maintain their cognitive abilities for longer, due to having higher cognitive reserve (Barulli et al., 2013; Dubois et al., 2016; Kaufman et al., 2016), resulting in increased mental capacity to compensate for negative age-associated changes. Furthermore, in a portion of older adults, labelled as super agers, their level of cognitive capacity remains constant throughout adulthood and as such these individuals perform comparably with younger individuals (Glisky, 2007; Cadar, 2018; Zanto and Gazzaley, 2019). However, in some older adults, the rate of cognitive decline can be substantially greater than expected and may signal the onset of a neurodegenerative condition. Therefore, a thorough understanding of the exact nature of cognitive decline in aging is relevant for clinical diagnosis and may, furthermore, provide the basis for developing and fine-tuning training regimes aimed at slowing the age decline (Bherer et al., 2005; Zinke et al., 2014; Mowszowski et al., 2016; Salminen et al., 2016; Penning et al., 2021).

Decline in cognitive abilities frequently reflects a decline in executive functioning (Engle et al., 1999; Craik and Salthouse, 2008; Urbanowitsch et al., 2015; Wongupparaj e al., 2015). These are traditionally conceptualized as a set of high-level cognitive processes implicated in the control and regulation of lower-level cognitive functions of goal-directed and future-oriented behaviors (Engle et al., 1999; Craik and Salthouse, 2008; Urbanowitsch et al., 2015; Wongupparaj et al., 2015). Miyake et al. (2000) deemed three EFs (response) inhibition, (mental) shifting, and working memory (WM) updating to be fundamental subcomponents of EF. They have been found to be commonly recruited in everyday activities and tasks (McAlister and Schmitter-Edgecombe, 2016; Li et al., 2019). Further analysis of a fourth component, i.e., dual-tasking, was not as clear. Miyake et al. (2000) reported that although these EFs are a heterogeneous group of functions, i.e., ‘*diverse’*, they share an underlying common factor, i.e., ‘*unity’*, finding that inhibition, shifting and updating loaded with similar factor, while dual-tasking loaded uniquely from the three. However, this finding was demonstrated in young adults only. Additional research on older individuals have reported different patterns of factor loadings (Hedden and Yoon, 2006; Hull et al., 2008; Vaughan and Giovanello, 2010; Bettcher et al., 2016; Bock et al., 2019; Glisky et al., 2020). In particular, Glisky et al. (2020) found updating and inhibition loaded on a common factor, whereas shifting loaded separately (dual-tasking was not assessed), and the loading (unity) was stronger in the even older group. Therefore, indicating an age effect and that EFs possibly decline at diverse rates.

Several theories have been proposed for the cause of cognitive decline. A popular one is the inhibition deficit theory (Hasher and Zacks, 1988), which proposes that older adults are more susceptible to the effects of distracting interference during the performance of cognitive tasks as a result of reduced attentional control (Lustig et al., 2007; Borella et al., 2011; Coubard et al., 2011; Tsang, 2013; Sylvain-Roy et al., 2015; Burda et al., 2017; Fountain-Zaragoza et al., 2018). Specifically, the ability to remove irrelevant information effectively is affected, limiting the retrieval of task-relevant information, which is thought to be due to a reduction and weakening of inhibitory resources with aging. The other theories include the prefrontal-executive hypothesis by West (1996), processing-speed theory by Salthouse (1996), executive attention framework (Engle, 2002; Engle and Kane, 2004), and strategy-deficit hypothesis (Bailey et al., 2009). The prefrontal-executive theory proposed by Dempster and Vegas (1992) and validated by West (1996) implies structural and functional changes in the prefrontal cortex (PFC) regions, as observed during aging and in neurodegenerative conditions, causes EF decline. This theory is in line with the fact that the PFC is assumed to be the prime neuroanatomical location of EFs. Other theories offer explanations regarding the subsequent performance of older adults. Salthouse (1996) theorized in the processing-speed theory that age-associated deficits in cognitive functioning are due to the reduction in the speed of processing operations leading to difficulties in the storage of information. It suggests processing is slowed because required actions cannot be successfully implemented, i.e., encoded, and stored within an adequate timeframe (limited time). Also, performance is affected because early processed information may not be available once processing is complete as the information is removed before it can be rehearsed or retrieved from storage (simultaneity) (Salthouse, 1996). The executive attention framework (Engle, 2002; Engle and Kane, 2004) states that older individuals are less efficient in their maintenance of attentional control when active tasks are experienced in difficult settings with high task-interference. This may cause problems in the planning and execution of complex tasks, as these individuals may be unable to focus on task requirements (Hasher and Zacks, 1988; Rubinstein et al., 2001; Sylvain-Roy et al., 2015; Bier et al., 2017). Lastly, the strategy-deficit hypothesis (Bailey et al., 2009) describes how the ineffective or deficient use of strategies in older adults leads to additional age-associated performance deficits. Specifically, in that they have difficulty producing and using appropriate strategies to encode information that may be required for task completion. Though these are separate theories, they are directly and indirectly linked to each other. Thus, cognitive aging may be viewed as a heterogeneous process through the involvement of various physiological and cognitive processes affecting cognition.

Dual-tasking, described as the simultaneous performance of two tasks (Della Sala et al., 1995; Baddeley, 1996; Miyake et al., 2000), has been shown to have age-related impairment (Craik, 1977; Wright, 1981; McDowd and Craik, 1988; Craik et al., 1996; Hartley et al., 1999; Verhaeghen and Cerella, 2002; Verhaeghen et al., 2003; Strobach et al., 2012; Fraser and Bherer, 2013). Older adults are reported to be able to complete such tasks but at a slower rate (Verhaeghen and Cerella, 2002). Typically, dual-tasking is assessed by the difference in response times (RTs) and error-rates produced in the single-task (ST) and the dual-task (DT) conditions, which is referred to as the DT costs. Several studies have described higher DT costs in older adults in comparison to younger individuals (Craik, 1977; Wright, 1981; Salthouse et al., 1984; McDowd and Craik, 1988; Crossley and Hiscock, 1992; Hartley et al., 1999; Hartley, 2001; Verhaeghen et al., 2003; Naveh-Benjamin et al., 2005; Craik and Salthouse, 2008). That is, the older individuals generally made much more errors and generated even longer RTs during the DT condition in comparison to the ST condition than younger individuals. This could be indicative of a reduction in processing resources as dual-tasking creates more competition for limited resources, such as attention, than ST situations. Bier et al. (2017) found with the utilization of an auditory digit span task and a visuospatial tracking task that older participants were incapable of controlling their attention, concluding it to be the significant factor for the age-related difference in DT costs. Although it has also been suggested that there is no age-associated decline in dual-tasking (Logie et al., 2004; Anderson et al., 2011; Argiris et al., 2019). However, overwhelming evidence confirms that decline in (divided) attention is a prominent factor affecting older adults in completing DTs.

Inhibition is defined as the intended inhibition of prepotent responses or suppression of dominant responses. It refers to the process by which automated, previously prepared responses are suppressed (Miyake et al., 2000; Salthouse et al., 2003; Diamond, 2013; Bender et al., 2016). Older individuals seem to be less proficient at efficiently suppressing irrelevant thoughts and actions which is believed to be linked to decreased attentional control (Wecker et al., 2000; Borella et al., 2008; Adólfsdóttir et al., 2017; Zuber et al., 2019), as theorized as the inhibition-deficit hypothesis (Hasher and Zacks, 1988; Lustig et al., 2007). However, a meta-analysis performed by Rey-Mermet and Gade (2018) suggested otherwise. Differences in inhibitory decline were observed with the utilization of different tasks (Rey-Mermet and Gade, 2018). For instance, no age-related deficit was reported with the Stroop (1935), flanker (Eriksen and Eriken, 1974), and global–local (Navon, 1977) tasks, but were with the go/no-go (Newman and Kosson, 1986), and stop-signal (Logan et al., 1984) tasks. The results were inconclusive for the Simon, and the positive and negative compatibility tasks. Due to these inconsistences, it is unclear whether older individuals are indeed less effective in inhibition. However, it may be that only certain tasks are able to highlight the issue, or that “inhibition” is not one unitary concept but has different aspects, e.g., response inhibition vs. perceptual inhibition, and that only some of those aspects are affected.

Similar to inhibition, it is unclear if the ability to maintain and coordinate two alternating task sets, i.e., shifting or switching (Miyake et al., 2000; Fisk and Sharp, 2004; Diamond, 2013; Schnitzspahn et al., 2013), is affected by aging. Verhaeghen and Cerella (2002) determined in their meta-analysis that it did not show a specific age-related deficit. A conclusion which was also reported Zuber et al. (2019). Nonetheless, in another meta-analysis by Wasylyshyn et al. (2011) and a paper by Verhaeghen (2011), a deficit in shifting was reported for the global shift cost only (the difference in shift RTs and error-rates from shift blocks and repetition RTs and error-rates from repetition blocks). Local shift costs (the differences in RTs and error-rates between non-shift and shifting trials within shifting blocks – please refer to the task switching task description for more detail) were considered comparable between the age groups. However, local shift costs are typically referred to as the better measure of shift costs, thus it may be concluded that no deficit was observed. Adólfsdóttir et al. (2017) also reported age effects in shifting costs in their longitudinal study but they failed to indicate the shift type and acknowledged that they did not analyze the error-rate effects. Furthermore, the task employed can affect the result observed, i.e., the level of impairment may differ. Wecker et al. (2005) reported differences in the nature and size of age-related shifting decline with the use of three tasks, the trail making test (TMT), verbal fluency test and design fluency test, which require verbal and nonverbal cognitive shifting. Therefore, it is uncertain as to whether aging does affect shifting and/or if the sensitivity of the task employed determines the level of decline observed in this population.

The process of WM updating is described as the constant revision of information in short-term memory, and the monitoring of WM by assessing ongoing functions and detecting errors (Engle et al., 1999; Miyake et al., 2000; D’Esposito and Postle, 2015). WM updating has been reported to undergo moderate decline with age (Zuber et al., 2019). With the use of a letter span task, Van dar Linden et al. (1994) observed that with low memory-load demands, older participants were comparable to younger individuals in their performance. However, with high memory-load demands, older participants’ updating capability was affected, and concluded to be the result of deficits with the processing resources of the central executive. A finding that was confirmed by Artuso et al. (2017). Similarly, a steady decline in WM updating ability with age has been observed with the utilizing of the backward digit span (BDS) task (Grégoire and Van der Linden, 1997). In line with this, De Beni and Palladino (2004) also reported older individuals had more difficulty in recalling spans. With span tasks, it seems a decline in updating ability is attributed to an increase in intrusion errors, caused by a failure to eliminate previously activated irrelevant information in older adults (Palladino and De Beni, 1999; De Beni and Palladino, 2004). Further, age-related decline with the use of another updating task, the n-back task was shown in a meta-analysis by Bopp and Verhaeghen (2018) where older individuals performed worse with longer lists, particularly if *n* is larger than 1 (i.e., n = 2, n = 3). Accordingly, these studies suggest that updating is affected by the aging process.

Having discussed the four EFs individually, it may be concluded that they all appear to be affected by the aging process to a varying degree. However, it is unknown whether they are affected in the same way, or if some are more affected than others. In the present study, the individual decline rates of these EFs were explored as a consequence of aging. We used a mixed design, with the between-subject factor Group (cognitively healthy young vs. cognitively older adults) and the within-subject factor EF (inhibition, shifting, updating, dual-tasking; each EF was assessed by two separate tasks). For all tasks and participants, we calculated cost measures by subtracting a condition with no or low EF demands from an EF demanding task condition (e.g., subtracting task repetition performance data from task shifting performance data, or subtracting ST from DT performance) where possible. We hypothesized that the older adults would show higher costs in measures of speed and accuracy than the young adults. Based on the above discussions, we speculated that age-related decline would be highest for updating, followed by inhibition, then dual-tasking, and smallest for shifting. We calculated correlations among all tasks and hypothesized that correlations among tasks aiming to assess the same EF construct would be higher as compared to correlations with tasks assessing other EF constructs. We further hypothesized more correlations in the older adults due to the change of EF structure with age.

## Materials and methods

### Participants

31 (7 M/24F) young adult participants were initially recruited into the study, three (2 M/1F) withdrew after the first (screening) session and two (2F) after the second. The data for the two individuals who completed only two of the three sessions was used in the study. Thus, 26 participants (5 M/21F), aged 18 to 33 years (mean: 21.18, SD: 4.43), completed all study sessions. In addition, 25 older adult participants (11 M/14F) aged 60 to 84 years (mean: 71.56, SD: 6.63), were recruited into the study. There were no withdrawals. All participants had normal or corrected to normal vision and hearing, and participants had no history of neurological diseases or took any medication that may affect cognition. The study was approved by Brunel University’s Life Sciences Ethics Committee and conducted in accordance with the Declaration of Helsinki.

### Procedure

The young adult participants were recruited through poster and online advertisement at Brunel University London, and the older adults through poster placement in the Brunel Older People’s Reference Group (BORG) newsletter, online advertisement on the university intranet, by handing out leaflets to the public, and by word of mouth.

Once a participant gave written informed consent, they completed an online study-recruitment questionnaire to determine their suitability for study participation in their own time prior to any study session. Data collected included demographic information, level of education, profession, medical history of severe auditory or visual abnormalities, and psychiatric, neurological, or systemic diseases which could cause cognitive impairments. In addition, severe physical disability, a history of epilepsy or other conditions that may cause uncontrolled movements or tremors were all considered exclusion criteria. Once accepted for participation, all individuals were invited to the screening session.

Participants completed three sessions, the screening session and two EF-sessions, each lasting approximately 60 min in duration. With the older adults, most study visits took place at the participant’s home. With the remaining participants, sessions were completed at Brunel University London’s Uxbridge campus or at a local library or facility of the participant’s choice. The participants completed the tests, the Montreal Cognitive Assessment (MoCA) (Nasreddine et al., 2005), Mini-Mental State Examination (MMSE) (Folstein et al., 1975), geriatric anxiety scale (GAS), geriatric depression scale (GDS) (Yesavage et al., 1983), activities of daily living scale (ADL) instrumental activities of daily living scale (IADL), and the spot-the-word test online *via* a Qualtrics link. The Hopkins verbal learning test (HVLT) (Brandt, 1991) was completed in person.

In the first EF session, the assessments were completed in the following order: test for everyday attention (TEA) DT telephone code search subtest, computerised task switching test, backward digit recall span (BDS), and the Hayling sentence completion test (HSCT). In the second EF session, the assessments were completed in the following order: trail making test (TMT), computerised n-back, Stroop task, and the computerised psychological refractory period (PRP) paradigm task. All the computerised tasks except the BDS and Stroop task, included a practice run prior to beginning the actual study task.

Participants completed the sessions in the same order as outlined above. However, due to computer issues, two older participants did not follow this order. Following completion of all study sessions, all participants were debriefed and compensated with either 12 course credits for the undergraduate psychology students or a £20 Amazon voucher.

### Screening assessments

The following tests measured the cognitive function and premorbid intelligence level of the participants during the screening session (Session 1):

#### Montreal cognitive assessment

The MoCA is a pen-and-paper screening instrument used to detect cognitive decline and takes approximately 10 min to administer. It consists of eight domains: visuospatial/executive function, naming, memory, attention, language, abstraction, delayed recall, and orientation. Participants were assessed in an interview type setting with an examiner (all assessments were conducted by Mojitola I Idowu). Scores range from 0 to 30 and are based on accuracy performance. Scores greater than 25 suggest normal cognition, 20 to 25 mild cognitive impairment (MCI), 19 to 14 early-stage dementia, and below 14 indicate dementia (Nasreddine et al., 2005).

#### Mini-mental state examination

The MMSE tool is a pen-and-paper screening instrument used to detect cognitive decline and takes approximately 10 min to administer. It is a simple test of cognitive function and is based on a total possible score of 30 points. It is divided into six domains: orientation, concentration, attention, verbal memory, naming, and visuospatial skills. A score of 28–30 suggest normal cognition, 25–27 MCI, 19–24 mild dementia, 10–18 moderate dementia, 0–9 indicates severe dementia (Folstein et al., 1975).

#### Geriatric anxiety scale

The GAS is a self-report anxiety measure specifically developed for use with older adults and takes approximately 5–10 min to complete. It consists of 30 items of which 25 items represent three common domains of anxiety symptoms among older adults (cognitive, somatic, and affective) and the last 5 items represent common content areas of worry. There are approximately 8 to 9 items for each domain. Participants are required to indicate how often they have experienced each symptom within the last week and including the current day of the assessment using the 4-point Likert scale ranging from 0 (not at all) to 3 (all of the time). Higher scores indicate higher levels of anxiety. Scores are generated from the 25 items of the three common domains only, obtained by summing the point values assigned to each response. The additional 5 are for clinical use only. Thus, scores can range from 0 for no anxiety to 75 for severe anxiety. This test was completed online *via* a Qualtrics link (Segal et al., 2010).

#### Geriatric depression scale

The GDS is a self-report measure consisting of 30 yes/no response questions designed specifically for assessing depression in older adults. It takes approximately 5 to 10 min to complete. Scores are generated from the summation of the first 25 items, where responses are designated either a ‘0’ or ‘1’. Higher total scores indicate a higher level of depression. The remaining 5 items are for clinical use. The following cut-off points are used to determine depression level: 0–9 normal range; 10–19 declares mild depression; 20–30, moderate to severe depression. This test was completed online *via* a Qualtrics link (Yesavage et al., 1983).

#### Activities of daily living scale

The ADL is a 6-item test assesses an individual’s present level of functional ability on a series of basic activities performed daily required for independent living at home and/or in the community. These functions include personal, self-care, domestic and general home maintenance activities in and around the home. Scores are out of 6, with 6 representing the best level of independence. It takes approximately 2–5 min to complete. This test was completed online *via* a Qualtrics link (Lawton and Brody, 1969).

#### Instrumental activities of daily living scale

The IADL is an 8-item test that assesses slightly more complex skills not fundamental to life than the ADL, however aid in an individual’s ability to live independently in a community. These skills include managing finances, handling transportation, shopping, preparing meals, using the telephone or other communication devices, managing medications, doing laundry, housework, and basic home maintenance. Scores are out of 8, with 8 representing the best level of ability. It takes approximately 2–5 min to complete. This test was completed online *via* a Qualtrics link (Lawton and Brody, 1969).

#### Spot-the-word test

This test is used to estimate premorbid intelligence which takes approximately 10 min to complete. It involves presenting the participants with pairs of items comprising one actual word and one non-word (e.g., *lentil* or *glotex*) and requiring the participant to identify the actual word. The participant’s number of correctly identified true words was recorded out of a total of 60. This test was completed online *via* a Qualtrics link (Baddeley et al., 1993).

#### Hopkins verbal learning test

The pen-and-paper HVLT is a brief measure of verbal memory which takes approximately 10 min to administer. In part A, the immediate recall section, participants are read a list of 12 words composed of four words from 3 semantic categories (e.g., ‘precious stones—emerald’; ‘human shelter - hotel’; ‘animals—tiger’) aloud and asked to recall as many as they can by the examiner. This is repeated three times. An average score is derived from the total of the 3 free recall trials (‘Total recall’). Part B, delayed recall section, involved the recognition of words from part A, where a single list of 24 words was read aloud by the examiner and participants were asked to identify which of these words were included in the original list of 12 by responding with ‘yes’ or ‘no’. This was performed once. Correctly identified words were recorded as true positives. The list also contained 6 distractors from the same semantic categories (related false positives or FP-related) and 6 unrelated distractors (unrelated false positives or FP-unrelated). The Discrimination index (true positives - false positives) was calculated (Brandt, 1991).

### Executive function assessments

Each EF was assessed with two individual tasks to examine the cognitive ability of the participants in dual-tasking, inhibition, shifting and updating.

#### Dual-tasking

##### Pen-and-paper based modified test for everyday attention DT telephone code search subtest

The DT telephone code search subtest assesses divided attention. Participants were required to undertake a visual search for several occurrences of a specific telephone area code with a corresponding symbol in a fictional telephone directory consisting of several telephone area codes with corresponding symbol combinations. At the same time, participants were additionally required to count the number of low tones they heard in a series of randomly mixed low and high tones played aloud by a computer or laptop in the background. They were given 2 min to complete this. The participant’s count of area code/symbol and low tone audio number were recorded. There were 17 target phone numbers in the list, and 10 low tones (Robertson et al., 1994).

The older adult participants performed both tasks individually as ST prior to undertaking the DT condition. Hence, they counted a series of auditory tones being played in the background as a ST and searched for a specific area code with a corresponding symbol in a fictional telephone directory, as another ST. The correct auditory number was 4 and telephone code count was 7.

#### Psychological refractory period paradigm task

The PRP task ran in Presentation (version 18.1.06.09.15^1^). Participants were required to give two responses (R1 and R2) to two stimuli (S1 and S2), auditory and visual, separately as STs and concurrently as DTs in separate blocks. Each block was cued at the start, so participants were aware of which task to perform (Pashler, 1984).

##### Single-tasks

In the auditory ST, participants were required to discriminate between high and low tones during 2 blocks of 25 trials each. Each tone sequence was played, while a black computer screen was displayed for 300 ms. On a QWERTY keyboard ‘Z’ and ‘X’ keys represented ‘low’ and ‘high’ frequency tones, respectively. Similarly, in the visual ST, participants are required to discriminate between the numerical values ‘1’ or ‘2’ during 2 blocks of 25 trials each. On a QWERTY keyboard, the keys ‘N’ and ‘M’ represented ‘1’ and ‘2’, respectively. Each presentation occurs on a black computer screen for 300 ms. In both cases, the participant was required to respond within 9,000 ms otherwise an error was recorded. Participants were instructed to respond using their index finger of each hand during both ST condition. The overall task duration was dependent on the response speed of the participant. Performance was assessed by the average RTs and error-rates produced for each ST.

##### Dual-tasks

In the DT condition of the PRP task, participants were required to complete 4 blocks of 25 trials, 2 for each of the two stimulus onset asynchronies (SOA), 0 ms and 1,000 ms, where the auditory stimulus was always presented before the visual stimulus.

In both SOAs, participants were required to respond to the auditory stimulus first and then to the visual stimulus within 9,000 ms, otherwise an error is recorded. Each stimulus was presented on a black screen. Participants responded with the same keys as in the ST, however, used their index and middle fingers from each hand to select between ‘Z’ and ‘X’, and ‘N’ and ‘M’. The overall task duration was dependent on the response speed of the participant. Performance was assessed by the average RTs and error-rates produced for each DT condition, i.e., SOA 0 ms and SOA 1000 ms. DT cost (DT minus ST performance) and the PRP effect (DT SOA 0 ms minus DT SOA 1000 ms performance) were also calculated for the RTs and error-rates at SOA 0 ms.

#### Inhibition

##### Stroop task

The pen-and-paper Stroop task was administered to measure the participants susceptibility to Stroop interference and took approximately 5 min to complete. This task consisted of three parts, each having 100 items organized in five columns of 20. Part 1, word reading (W), had the words **RED**, **GREEN**, and **BLUE** printed in black ink, randomly arranged. No word followed itself within a column. Part 2, color naming (C), had items written as XXXX in the colors green, blue, and red, i.e., **XXXX**, **XXXX** and **XXXX**, randomly arranged. No color followed itself or matched the corresponding item. Part 3, naming of the incongruent color of the ink of the word presented, color-word (CW), consisted of the words in part 1 printed in the color of the items in part 2 for all 100 items, e.g., **RED**, **GREEN**, or **BLUE**, randomly arranged. This section assessed the inhibition ability of the participant to inhibit the word presented as reading is an automated process whilst color naming is not. Participants were given 45 s for each part and instructed to read the words, name the colors, and name the ink color of the printed words, respectively, as quickly and as accurately as possible. The number of correctly read words or colors out of 100 was recorded for each section, i.e., W, C and CW (Golden, 1978).

In addition, following completion on the task, the predicted CW, which is based on the individual’s performance in the W and C sections, denoted as CW’, was calculated using the formula indicated, (W × C) / (W + C). Inhibition ability is based on the interference score, calculated with the formula stated, CW – CW’.

##### Hayling sentence completion test

Participants were required to provide either the expected word or an unrelated word to complete a high cloze sentence. The test consisted of two parts, each with a set of 15 sentences with the last word missing; for example, ‘*The captain wanted to stay with the sinking...*’. In part 1, participants had to produce a word that best finished each sentence (Initiation condition), e.g., “ship.” In part 2, participants had to complete each sentence by inhibiting an impulse to give the word that best completed the sentence by instead giving an unconnected word (Inhibition condition), e.g., “colour.” A prerecording of all the sentences was played to the participants and paused whilst they were being audio recorded for their verbal answers to be collected. Immediately after a participant gave a response, the next sentence was played. The overall task duration was dependent on the speed of the participant in producing a word for all the sentences but was usually between 7 and 10 min. Performance was measured by the total time taken to produce the words in each part, and the incorrectness of the words to the sentences in part 2. Furthermore, the task generates its own derived performance score by adding the scaled score of the RTs of the two sections, and the errors produced in the incongruent section, part 1 scaled score + part 2 scaled score + part 2 errors scaled score. Part 2 errors were scored as ‘any category A (connected, related word) errors’ + ‘any category B (somewhat connected word) errors’ and then scaled using the appropriate task table (Burgess and Shallice, 1997).

#### Shifting

##### Task switching test

In this computerized task, ran in Presentation software (version 18.1.06.09.15, www.neurobs.com), participants were required to perform two test conditions, a repetition, and a shifting, where the stimulus, a ‘1’ or ‘2’ printed in the colors blue or yellow, respectively were presented. In the repetition condition, participants had to complete 2 blocks of 30 trials of two separate cued conditions, a numerical and color, hence 4 blocks in total. In the cued numerical condition, the participants had to decide whether a ‘1’ or ‘2’ was presented, and in the cued color condition whether the color of the number was ‘blue’ or ‘yellow’. On a QWERTY keyboard the ‘Z’ key represented ‘1’ and ‘blue’, and ‘M’, ‘2’ and ‘yellow’. Instructions were displayed on the screen at the start of each block until the participant started the task. The stimuli were presented on a black screen for 300 ms until the participants responded or timeout after 9,000 ms from stimulus onset, where an error was recorded. Participants were instructed to respond using their index finger of each hand (Rogers and Monsell, 1995).

In the task switching condition, the two repetition conditions were mixed and presented randomly within a block. Participants were cued as to which task to perform next, e.g., number or color through the presentation of instructions on the screen at the start of each trial until the participant started the task. The cue-target interval (CTI) was 0 ms. There were 30 mixed trials per block and 4 blocks of the shifting task. Each stimulus was presented on a black screen for 300 ms until the participants responded / timed out, where an error was recorded.

The repetition and shifting blocks were presented in a pseudo-randomized order in the test. The overall task duration was dependent on the response speed of the participant. Performance was assessed by the average RTs and error-rates produced during the repetition and shifting conditions. The shifting cost was then determined using the formula ‘shifting – repetition’ for both the RT and error-rate, where smaller values represent better shifting performance. The local shift (difference between shifting and non-shifting trials within a shifting block), global shift (difference between the shifting blocks and the pure repetition blocks), and mixing-cost (difference between the repetition trials within the pure repetition blocks and within the combined shifting and repetition blocks) were assessed.

##### Trail making test parts A and B

The pen-and-paper TMT for cognitive flexibility encompassed two parts, A and B, consisting of 25 small open circles randomly distributed over a sheet of paper. In TMT part A, the circles are numbered 1 to 25, and the participant is asked to draw lines to connect them in ascending order as quickly as possible. In TMT part B, the circles include both numbers (1 to 13) and letters (A to L). The participant must draw lines to connect the circles in ascending order as quickly as possible while alternating between the numbers and letters (i.e., 1, A, 2, B, etc.,). The completion time and the number of errors were recorded. Data from this task were excluded when participants made more than 2 mistakes in either part. The overall task duration was dependent on the completion times of both parts by the participant. The shifting cost was then determined using the formula ‘shifting (TMT part B) - repetition (TMT part A)’ for the RT, where smaller values represent better shifting performance. Similarly for error-rates, 2 errors were equated to an error-rate of 100% in each part, hence the error-rate cost was also determined (Reitan, 1992).

#### Updating

##### N-Back test

In this computerized spatial task, ran in Presentation software (version 18.1.06.09.15, www.neurobs.com), participants were instructed to indicate the position of the stimulus, a yellow circle, presented to them on a black computer screen. The task involved 4 conditions where the participant must respond with the position of the yellow circle seen at *n* screens back to the present screen. The *n* positions used in this research were 0 (the present screen), 1-, 2-, and 3-back screens prior. Participants were instructed to select the corresponding QWERTY keyboard button they thought was the position of the circle, i.e., ‘V’ for the first (left-most) position, ‘B’ for the second, ‘N’ for the third and ‘M’ for the fourth. They could respond using their index and middle fingers of each hand or four fingers from a preferred hand (Kirchner, 1958).

The targets (i.e., the yellow circles) were presented on a black computer screen with a white horizontal “grid” consisting of 4 boxes of the four possible locations of the circle for 2000 ms or until the participants responded, otherwise it was recorded as an error. There were 5 blocks for each n-back condition, i.e., 20 blocks in total. One block consisted of 16 trials, so 320 trials in the whole experiment. Conditions were always presented in the fixed order, blocks 1–5: 0-back, blocks 6–10: 1-back, blocks 11–15: 2-back, and blocks 16–20: 3-back. Instructions were presented on the screen at the start of each block until the participant started the task. Thus, a trial took a maximum of 2,750 ms in the case of time-out, or shorter if responded before the time-out.

Participants were required to indicate at what position on the screen the yellow circle appears by selecting an appropriate button on the keyboard. In the 0-back condition, the position of the circle on the present screen is recorded for all trials within the block. In the 1-back, the participant must respond to the circle’s position on the previous screen, in the 2-back, its position two screens before, and in the 3-back, three screens prior. Performance was assessed by the average RTs and error-rates produced during each n-back condition. Analysis was then conducted by comparing the n-back conditions among each other, e.g., 0 vs. 1, 0 vs. 2, 0 vs. 3, 1 vs. 2, etc., with the RTs and error-rates.

##### Backward digit recall span

In the pen-and-paper BDS, participants were required to recall a list of numbers in reverse order immediately following presentation from a pre-recording on a computer or laptop. The examiner paused the recording after each list to allow the participant to respond. The minimum length of the list is 2 and maximum 8, and there are two trials per length. The longest correct list of numbers the participant can recall backwards once is recorded as a measure of their WM capacity. Performance assessment is determined by scoring ‘1’ for each correctly recalled length, thus ranging 1 to 14, e.g., if all trials are performed correctly then the score would be ‘14’ for 2 trials for all 7 lengths (Baddeley and Hitch, 1974).

### Statistical analysis

#### Cross-sectional study

Sociodemographics, screening assessments, and the EF task measures were compared between the age groups using independent *t*-test or analyses of variance (ANOVA), and chi-squared, χ^2^ test for the categorical variables gender and handedness. For the EF task comparison analysis, the cost measures were primarily used. Examples of such measures include the DT costs (DT minus ST condition measures), inhibition costs (incongruent minus congruent), shifting costs (shifting minus repetition condition measures) (Wylie and Allport, 2000), updating cost (3-back minus 0-back). Analyzing the difference between simple and complex task conditions eliminates unwanted cognitive and nonexecutive processes. To test for group differences, we assumed a 2 × 2 factorial mixed design, with the within-subject factor Task (simple vs. complex task, e.g., DT vs. ST) and the between-subject factor Group (young vs. old). The interaction term of this ANOVA is of particular importance as it reflects whether the difference between simple and complex tasks (i.e., the costs) differ between the groups. For some variables, e.g., the derived test scores of the Stroop and HSCT tasks calculated according to the respective test manuals, we used independent *t*-tests to compare the performance between the groups. Paired-sampled *t*-tests was further used to compare each n-back condition with one another.

Participants were excluded from analysis on each test if they performed above or below 3 standard deviations (SDs) from the rest of the group’s mean performance. Also, participants who produced an error-rate of 60% or greater in either condition of the TEA test and 50% or greater in either task condition of the PRP tasks were removed from the respective analysis. It was questioned if these participants understood the task requirements and/or performed the task properly.

#### Age-related decline in executive functions

To compare the age-related cognitive decline across the four EFs, selected performance measures of each task were z-transformed and averaged (see Results for details). Independent *t*-tests were conducted between the young and older adult scores. To determine how similar the age-related decline rate was between the age groups, the largest average task score for each EF was tested against each other by calculating factorial ANOVAs.

#### Correlation analysis

Correlation analysis was conducted to test how similar or different the respective task measures of the same and different EF constructs were. This was performed between the averaged z-score measures of each EF task and all z-score task measures of the young and older adult, separately using Pearson’s correlation, *r*, coefficient.

#### Effect size and level of significance

All the analyses except the correlation included the effect size, Cohen’s *d*, or partial eta squared, *Ƞ_p_^2^*. Effects were classified as small, if less than or equal to 0.2, moderate, if above 0.2 and less than or equal to 0.5, large, if above 0.5, and less than or equal to 0.8, and very large if greater than 0.8 (Cohen, 1988).

Significance was determined with *p* < 0.05. Bonferroni correction was also performed for each EF construct to adjust the false-positive rate and reported. Therefore, statistical significance was considered for dual-tasking 0.003 (0.05/20), for inhibition 0.005 (0.05/11), for shifting 0.002 (0.05/24), and for updating 0.005 (0.05/11). The updating *value of p* was further adjusted for the six n-back pairwise comparison analysis.

## Results

### Demography and screening data

As required in this cross-sectional study, a significant age difference was observed between the two groups *t*(51) = −32.83, *p* < 0.001, *d* = 9.19, please see Table 1 for all the results discussed in the section. They had comparable levels of education, *t*(50) = −0.80, *p* = 0.425, and showed no difference in the assessment of estimated premorbid IQ with the utilization of the spot-the-word test, *t*(51) = 0.43, *p* = 0.671. The MMSE and MoCA tests were used to determine the cognitive status of all participants. Both groups average scores suggested normal cognitive status and comparable status, the MMSE score, *t*(51) = −0.45, *p* = 0.651, *d* = 0.13, and the MoCA score, *t*(51) = −0.15, *p* = 0.885, *d* = 0.04.

**Table 1:** Demographic data and results of the screening tests of the young and older adult participants.

Characteristic	Young adults (mean/SD)	Older adults (mean/SD)	*t/ꭓ* ^2^	df	Young vs. old, *p-*value
Age (years)	21.18 (4.43)	71.56 (6.63)	−32.83	51	**<0.001** ^**b**^
Gender (M/F)	5/23	11/14	4.28	1	**0.038** ^**c**^
Education (years)	14.46 (1.32)	14.68^a^ (2.08)	−0.80	50	0.425^b^
Handedness (L/R)	2/26	2/23	0.01	1	0.906^c^
Mini-Mental State Examination (min 0 – max 30)	28.46 (1.29)	28.64 (1.52)	−0.45	51	0.651^b^
Montreal Cognitive Assessment (min 0 – max 30)	26.71 (2.27)	26.80 (2.00)	−0.15	51	0.885^b^
Geriatric Anxiety Scale (min 0–75)	19.75 (9.99)	7.12 (5.49)	5.61	51	**<0.001** ^**b**^
Geriatric Depression Scale (min 0–30)	12.32 (3.40)	17.92 (1.47)	−7.62	51	**<0.001** ^**b**^
Activities of daily living scale (poor 1–6)	6.00 (0.00)	5.80 (0.41)	2.60	51	**0.012** ^**b**^
Instrumental activities of daily living scale (poor 1–8)	7.18 (1.28)	7.92 (0.28)	−2.84	51	**0.006** ^**b**^
Hopkins Verbal Learning Test, part A (min 0–12)	7.38 (1.63)	7.47 (1.62)	−0.19	51	0.849^b^
Hopkins Verbal Learning Test, part B (min 0–12)	11.07 (1.18)	10.28 (1.43)	2.20	51	**0.032** ^**b**^
Hopkins Verbal Learning Test, Discrimination index (min 0–12)	11.04 (1.23)	10.28 (1.43)	2.07	51	**0.044** ^**b**^
Spot-the-word test (min 0–60)	28.96 (3.11)	28.68 (1.25)	0.43	51	0.671^b^

a *n* = 24 as one individual’s educational level was unknown.

b Independent-samples *t*-test, *t*(51).

c Chi-squared test ꭓ^2^(1), *n* = 53.

Bold *p*-values indicate significant effects (*p* < 0.05).

Differences were reported in the quality-of-life assessments, ADL and IADL. The ADL test reported the young adults were better at completing everyday self-care tasks, such as bathing, dressing, and eating, *t*(51) = 2.60, *p* = 0.012, *d* = 0.73. Whereas for more complex daily tasks, including cooking, shopping, laundry, and housework, assessed with the IADL test, the older participants were better, *t*(51) = −2.84, *p* = 0.006, *d* = 0.80. These findings speak on the difference of life experience between the age groups performing such tasks, and the change in independence. Nonetheless, these differences did not affect the cognitive function required of them in completing this study.

In the evaluation of both groups’ anxiety and depression level, the GAS and GDS were employed. The GAS revealed that young adults showed significantly more anxiety than the older adults, *t*(51) = 5.61, *p* < 0 0.001, *d* = 1.57, however, both had fairly low mean score levels. With the GDS, the older participants were shown to have a moderate level of depression whilst the young adults presented with a low level, thus, a significant difference between the groups was also observed, *t*(51) = −7.62, *p* < 0.001, *d* = 2.13. No participant was reported to have been clinically diagnosed (e.g., by a medical profession) with anxiety or depression.

Verbal learning and memory were examined with the HVLT. A 2 × 2- factorial mixed ANOVA with the factors Group (young adults vs. older adults) and HVLT (part A, part B) indicated no main effect of Group, *F*(1, 51) = 1.07, *p* = 0.306, *Ƞ_p_^2^* = 0.02, and a significant main effect of the test part, *F*(1, 51) = 218.07, *p* < 0.001, *Ƞ_p_^2^* = 0.81, as part B, the recognition section, was performed with less accuracy. There was no interaction, *F*(1, 51) = 3.97, *p* = 0.052, *Ƞ_p_^2^* = 0.07. In sum, there were no statistically significant differences between the groups.

In conclusion, all scores represented normal functional ability and within normal cognition function ranges. There were insignificant differences between the groups’ cognitive statuses as observed by the MMSE and MoCA scores, and education level. Likewise, performance in the spot-the-word and HVLT were comparable. There was a difference in the level of anxiety and depression, and quality of life. Nonetheless, the groups’ cognitive ability was deemed equivalent and ideal for the comparison study detailed here.

### Dual-tasking

Dual-tasking ability was first examined with the TEA DT telephone code search subtest. Twenty-six young adults and 25 older adults completed the task, however, the data from 4 older adults was excluded as outliers. In addition, the data of two young adults and one older adult were not analyzed as these participants produced an error-rate of 60% or higher in either condition of the test. Please note, the young adult participants performed the tasks as DT only, i.e., no ST condition of the auditory and telephone code count tasks were completed, whereas the older adults completed the tasks as ST and DT, hence DT cost was not assessed, please see Table 2; Supplementary Table S1 for the performance measures. Instead, performance of the two tasks during DT performance was analyzed between the age groups. No age-related decline in the accuracy performance of either DT condition was found, auditory task *t*(42) = −0.55, *p* = 0.582, *d* = 0.17, and the DT telephone code search, *t*(42) = 0.72, *p* = 0.479, *d* = 0.22.

**Table 2:** Executive function abilities: cross-sectional analysis between the young and older adults.

Task	Young adults			Older adults			*t*	df	Young vs. old, *p*-value
	*N*	*O*	Mean (SD)	*N*	*O*	Mean (SD)			
Dual-tasking									
^1^ TEA Auditory DT count accuracy (%)	24	2	90.42 (13.34)	20	4	92.50 (11.18)	−0.55	42	0.582
^1^ TEA Telephone code count DT accuracy (%)	24	2	79.17 (15.12)	20	4	75.88 (15.26)	0.72	42	0.479
^2^ PRP Auditory RT1 (SOA 0 ms) DT (DT – ST) cost (ms)	24	0	462.65 (323.27)	22	3	439.85 (245.96)	0.27	44	0.790
^2^ PRP Visual RT2 (SOA 0 ms) DT (DT – ST) cost (ms)	24	0	809.45 (381.28)	22	3	905.37 (292.60)	−0.95	44	0.347
^2^ PRP effect (DT SOA 0 ms – DT SOA 1000 ms), visual task, RT2 (ms)	24	0	569.56 (254.83)	22	3	732.51 (250.94)	−2.18	44	**0.034**
^2^ PRP Auditory R1 (SOA 0 ms) DT (DT – ST) cost (%)	24	0	3.42 (6.92)	22	3	0.09 (3.53)	1.53	44	0.134
^2^ PRP Visual R2 (SOA 0 ms) DT (DT – ST) cost (%)	24	0	4.33 (5.71)	22	3	1.73 (2.41)	1.98	44	0.054
^2^ PRP effect (DT SOA 0 ms – DT SOA 1000 ms), visual task, R2 (%)	24	0	−1.33 (9.60)	22	3	1.09 (2.20)	−1.16	44	0.254
Inhibition									
^3^ HSCT inhibition RT cost (s)	26	1	6.77 (17.80)	23	1	28.30 (21.10)	−3.88	47	**<0.001**
Stroop interference score	26	0	6.92 (10.48)	25	0	−5.60 (7.71)	4.84	49	**<0.001**
Shifting									
^4^ TS Local shift RT cost (ms)	26	2	75.96 (84.29)	21	2	206.20 (239.41)	−2.58	45	**0.013**
^4^ TS RT Mixing-cost (ms)	26	2	286.67 (222.77)	21	2	404.14 (314.73)	−1.50	45	0.142
^4^ TS Global shift RT cost (ms)	26	2	363.06 (211.52)	21	2	619.79 (457.52)	−2.55	45	**0.014**
^4^ TS Error-rate TS Local shift cost (%)	26	2	4.77 (5.92)	21	2	3.10 (4.58)	1.06	45	0.293
^4^ TS Error-rate Mixing-cost (%)	26	2	0.88 (3.71)	21	2	4.81 (8.44)	−2.13	45	**0.038**
^4^ TS Error-rate Global shift cost	26	2	5.54 (5.59)	21	2	7.52 (9.14)	−0.92	45	0.364
^5^ TMT RT Shifting cost (s)	19	2	31.00 (17.68)	19	0	25.89 (11.99)	1.04	36	0.305
^5^ TMT error-rate shifting cost (%)	19	2	13.16 (36.67)	19	0	18.42 (47.76)	−0.38	45	0.705
Updating									
BDS score	28	0	7.86 (2.16)	25	0	7.68 (2.81)	0.26	51	0.797
N-back RT cost (ms)	26	0	25.88 (186.22)	23	1	6.34 (240.56)	0.32	47	0.751
N-back error-rate cost (%)	26	0	50.43 (14.34)	23	1	67.17 (14.52)	−4.06	47	**<0.001**

The table contains the independent *t*-tests of selected measures for all four executive functions between the young and older adults.

*N*, number of participants (after removal of outliers); *O*, number of all outliers (i.e., *N* + *O* = original sample size); BDS, Backward digit span task; HSCT, Hayling sentence completion task; PRP, Psychological Refractory Period paradigm; NB, N-back task; Stroop, Stroop task; TEA, Test for Everyday Attention; TMT, Trail making task; TS, Task switching task; RT, Reaction time; R, Error-rate; SOA, Stimulus Onset Asynchrony. Please note RT1 corresponds with R1, and RT2 with R2. Please note in addition to performance outliers (i.e., participants with mean scores outside +/− 3 SD of the group mean) the following were also excluded from analysis, ^1^ two young adults and one older adult TEA participants who produced an error-rate of 60% or higher in any of the task conditions, ^2^two young adults PRP participants who produced an error-rate of 50% or greater, ^3^one young and one older adult HSCT participants due to audio recording issues, ^4^ two older adults TS participants due to data recovery issues, and ^5^ one young and two older adults from TMT part A, plus 5 young and 4 older adults from part B who produced three or more errors in either task part. Bold *p*-values indicate significant effects (*p* < 0.05). (Bonferroni corrected alphas for dual-tasking *p* < 0.003, inhibition *p* < 0.005, shifting *p* < 0.002, and updating *p* < 0.005.)

Performance with the second DT, the PRP paradigm, was examined at SOA 0 ms and 1,000 ms, see Table 2 for the results. Twenty-six young and 25 older adults completed the task. However, two older adults were removed as outliers, while two young were not analyzed as they produced an error-rate of 50% or greater in one of the task conditions. Please note, in addition to the outliers, two young adult participants who produced an error-rate of 50% or higher in either task condition of the PRP tasks were removed from analysis. To assess performance between the age groups and the SOAs, the PRP effect, two 2 × 2- factorial mixed ANOVAs (one for RTs and one for error-rates) with the factors Group (young adults vs. older adults) and SOA (0 ms vs. 1,000 ms) were calculated for RT2, the visual stimuli, as it is the only stimuli presented at SOA 1000 ms (Pashler, 1994). For the mean RTs, there was no main effect of Group observed, *F*(1, 44) = 0.49, *p* = 0.486, *Ƞ_p_^2^* = 0.01. However, there was a main effect of the SOA, *F*(1, 44) = 304.07, *p* < 0.001, *Ƞ_p_^2^* = 0.87, as the SOA 1000 ms was completed faster. An interaction effect, *F*(1, 44) = 4.76, *p* = 0.034 (not significant following Bonferroni correction, alpha of 0.003), *Ƞ_p_^2^* = 0.10 was observed, thus, showing that the PRP effect was higher for the old participants. With the error-rate, there was no main effect of Group, *F*(1, 44) = 0.27, *p* = 0.608, *Ƞ_p_^2^* = 0.01, main effect of SOA, *F*(1, 44) = 0.01, *p* = 0.908, *Ƞ_p_^2^* = 0.000, or interaction, *F*(1, 44) = 1.34, *p* = 0.254, *Ƞ_p_^2^* = 0.03. Thus, there was a comparable mean error-rate cost generated by the PRP effect in the groups. Further analyses regarding differences between the STs and DT measures are available in the dual-tasking Results section in Supplementary material and comprehensive results in Supplementary Table S1.

To conclude, in the assessment of DT ability, age-associated decline was observed through its assessment with the RT of the PRP effect measure, although no significance was observed following Bonferroni correction. No age effect was found with the accuracy performance in the TEA DT telephone code search subtest.

### Inhibition

Inhibitory ability was examined first with the HSCT. Twenty-eight young and 25 older adults completed the task, however, one young and one older adult were excluded as outliers, and another young and older adult due to audio instrument failure as each participant was recorded. A 2 × 2–factorial mixed ANOVA with the factors Group (young adults vs. older adults) x HSCT part (part 1, part 2) for the RTs was conducted. A main effect of Group, *F*(1, 47) = 5.33, *p* < 0.001, *Ƞ_p_^2^* = 0.10, and a main effect for the HSCT part, *F*(1, 47) = 39.83, *p* < 0.001, *Ƞ_p_^2^* = 0.46, were observed. An interaction effect, *F*(1, 47) = 15.01, *p* < 0.001, *Ƞ_p_^2^* = 0.24, was observed, which indicated that the inhibition demand of the task affected the groups but to a different degree (Bonferroni corrected alpha of 0.005). Further analysis of the difference in the RT inhibition cost (part 2 minus part 1) between the age groups showed significance, *t*(47) = −3.88, *p* < 0.001, *d* = 1.13. The older adults showed larger inhibition costs than the young adults. Therefore, suggesting better performance of the young adults and indicating the older adults were affected more by the inhibition condition of the task. The task measures and additional analysis of the HSCT categorized scores are presented in Table 2 and Supplementary Table S2, including the additional Results section on inhibition in the Supplementary material, respectively.

Inhibitory ability with the Stroop task was assessed by the calculated interference score of the test (the actual CW minus the predicted CW’). Twenty-six young and 25 older adults completed the task, there were no outliers. The young adults scored 6.92 (10.48) and the older adults −5.60 (7.71). A positive calculated value indicates adequate ability in inhibiting interfering information, as seen with the young adults, whereas a negative interference value, shows poor inhibition ability, as seen with the older adults (Stroop, 1935; Scarpina and Tagini, 2017). This difference in interference score was statistically significant, *t*(49) = 4.84, *p* < 0.001, *d* = 1.38 (Table 2), showing a significant decline in inhibition ability with aging. Please refer to the Supplementary material for both groups’ performance data in Supplementary Table S2 and the inhibition additional Results section regarding analysis of the Stroop sections. In sum, analysis of inhibitory ability in the older adults with both tasks showed age-associated decline.

### Shifting

In the examination of shifting ability with the task switching task, three shifting cost types (shifting minus repetition condition measures) (Wylie and Allport, 2000) were assessed, local shift costs (difference between shifting and non-shifting trials within a shifting block), global shift costs (difference between the shift trials in shifting blocks and the pure repetition blocks), and mixing-costs (difference between the repetition trials within the shifting blocks and the repetition trials in pure repetition blocks). Twenty-eight young and 25 older adults completed the task, however, the data of two older adults was not used due to data recovery issues. In addition, two young adult and two older adult outliers were not analyzed. Analysis of the three costs described was completed through the calculation of 2 × 2–factorial mixed ANOVAs with the factors Group (young adults vs. older adults) x task switching part (repetition vs. shifting) for the RTs and error-rates.

The RT analysis revealed age-related decline in the local and global shift types. For local shift costs, a main effect of Group, *F*(1, 45) = 6.78, *p* = 0.012 (not significant following Bonferroni correction, alpha of 0.002), *Ƞ_p_^2^* = 0.13, a main effect of task condition, *F*(1, 45) = 31.43, *p* < 0.001, *Ƞ_p_^2^* = 0.41, and an interaction effect, *F*(1, 45) = 6.70, *p* = 0.013, *Ƞ_p_^2^* = 0.13, were observed. Global shift costs showed a main effect of Group, *F*(1, 45) = 8.63, *p* = 0.005 (not significant following Bonferroni correction), *Ƞ_p_^2^* = 0.16, a main effect of task condition, *F*(1, 46) = 115.87, *p* < 0.001, *Ƞ_p_^2^* = 0.72, and an interaction effect, *F*(1, 45) = 2.41, *p* = 0.128 (not significant following Bonferroni correction), *Ƞ_p_^2^* = 0.05. The significance of the interaction in both shifting costs showed that although both groups showed shifting costs, i.e., spend longer completing the shifting conditions, the older adults spent significantly longer in comparison to the young adults resulting in higher shifting costs. For the mixed block analysis in the task switching task, there was no main effect of Group *F*(1, 45) = 5.70, *p* = 0.021 (not significant following Bonferroni correction), *Ƞ_p_^2^* = 0.11, and a main effect of the task condition, *F*(1, 45) = 77.44, *p* < 0.001, *Ƞ_p_^2^* = 0.63. However, there was no interaction effect, *F*(1, 45) = 2.24, *p* = 0.142, *Ƞ_p_^2^* = 0.05, showing the effect of the task condition did not differ between the two groups.

With the error-rates there was an age-related cognitive decline in the mixed block analysis only. There was no main effect of Group, *F*(1, 45) = 0.19, *p* = 0.664, *Ƞ_p_^2^* = 0.004, a main effect of the task condition, *F*(1, 45) = 9.22, *p* = 0.004 (not significant following Bonferroni correction), *Ƞ_p_^2^* = 0.17, and an interaction effect, *F*(1, 45) = 4.46, *p* = 0.040 (not significant following Bonferroni correction), *Ƞ_p_^2^* = 0.09, indicating statistically significant differences in the accuracy performance between the repetition and shifting. For the local shift error-rate, there was no main effect of Group, *F*(1, 45) = 0.50, *p* = 0.484, *Ƞ_p_^2^* = 0.01, and a main effect of the task condition, *F*(1, 45) = 24.00, *p* < 0.001, *Ƞ_p_^2^* = 0.35, revealed as the shifting condition was completed with less accuracy. There was no interaction effect, *F*(1, 45) = 1.29, *p* = 0.263, *Ƞ_p_^2^* = 0.03, shown. For global shift error-rate, there was no main effect of Group found, *F*(1, 45) = 0.05, *p* = 0.825, *Ƞ_p_^2^* = 0.001, a main effect of task condition, *F*(1, 45) = 20.86, *p* < 0.001, *Ƞ_p_^2^* = 0.32, as the accuracy performance of the two conditions was not comparable, and no interaction effect observed, *F*(1, 45) = 3.13, *p* = 0.084, *Ƞ_p_^2^* = 0.07. Therefore, the older adults were especially affected by the demand of the shifting EF, producing higher RT costs during the local shift and global shift analyses, and a larger error-rate cost during the shifting block analysis. More performance data is available in Table 2 and Supplementary Table S3.

Twenty-six young and 25 older adults performed the TMT, however, only the participants that completed the entire test correctly, i.e., made no more than two errors in either part of the test were assessed, performance data is available in Table 2 and Supplementary Table S3. Hence, one young and two older adults were not assessed in part A, as well as 5 additional young and 4 older adults in part B. Another young adult was also excluded as a time outlier from part A. Performance was analyzed using two 2 × 2–factorial mixed ANOVAs with the factors Group (young adults vs. older adults) x TMT part (repetition vs. shifting) were calculated for the RTs and error-rates. For RT, no main effect of Group, *F*(1, 36) = 1.01, *p* = 0.321, *Ƞ_p_^2^* = 0.01, was found. There was a main effect of the TMT part, *F*(1, 36) = 134.75, *p* < 0.001, *Ƞ_p_^2^* = 0.79 due to the demand generated for shifting, but no interaction effect, *F*(1, 36) = 1.09, *p* = 0.305, *Ƞ_p_^2^* = 0.03, was shown. Therefore, the groups performed comparably in terms of the RT measure. All the performance data is available in Supplementary Table S3. For the error-rates, no main effect of Group was observed, *F*(1, 36) = 1.29, *p* = 0.264, *Ƞ_p_^2^* = 0.03. A main effect of the TMT part was found, *F*(1, 36) = 5.23, *p* = 0.028 (not significant following Bonferroni correction), *Ƞ_p_^2^* = 0.13 and no interaction effect, *F*(1, 36) = 0.15, *p* = 0.705, *Ƞ_p_^2^* = 0.004. Thus, no difference in the groups’ accuracy performance between the TMT parts was observed, which is understandable as these were the good performers. Therefore, with the restriction in error-rates it was not possible to detect any age-related differences in the accuracy performance of the TMT.

In conclusion, age-related decline in shifting ability was detected with the use of the task switching paradigm, in the local and global shift RT costs, and the error-rate mixing-cost but not following Bonferroni correction. With the TMT, insignificant performance differences were observed.

### Updating

In examining updating ability, performance in the BDS task was compared between the age groups where a total of 28 young and 25 older adults were assessed as there were no outliers. No age-related cognitive decline was observed, *t*(51) = 0.26, *p* = 0.797, *d* = 0.07. There was no difference in the span recall length as all the participants were able to recall up to 4 digits backwards (Table 2).

Twenty-six young and 25 older adults performed the n-back task, however, one older adult participant was excluded as an outlier. Age-related cognitive decline with the n-back task was assessed by calculating the updating cost measures (3-back minus 0-back) between the groups which revealed significance only in the mean error-rate costs, *t*(47) = −4.06, *p* < 0.001 (Bonferroni corrected alpha of 0.005), *d* = 1.18 [RT mean cost, *t*(47) = 0.32, *p* = 0.751, *d* = 0.09]. (Please note one older adult participant did not complete the 3-back condition). This reiterates the typical effect of this task on accuracy and not RT as a result of the strictly timed trial procedure. Additional analysis comparing the RTs and error-rates of all n-back conditions and their pairwise comparison between the groups is available in Supplementary Table S4 and the additional analysis in the updating Results sections in the Supplementary material, respectively.

To summarize, updating ability was comparable in performance between the two age groups with the BDS task. With the n-back task, however, a statistically significant difference was observed in the updating error-rate costs between the groups.

#### Age-related decline in executive function abilities

All performance output measures from all eight tasks were transformed into z-scores for both age groups. This allowed for comparison across different measures, such as costs and derived test scores. For the z-normalization, first the mean and standard deviation (SD) was calculated for each measure individually in the young adult group, so that after the z-transformation the young group had a mean of 0 and a SD of 1. Then these parameters (mean and SD) as determined in the young group were applied for the transformation of the older group, i.e., (older participant score – young adult mean)/young adult standard deviation. Consequently, the z-scores of the older adults reflected the amount of cognitive decline (or improvement) in terms of the young groups’ performance. Therefore, the mean z-scores of the older participants will not be zero, as would be observed with the young group. The results are listed in Table 3. (Please note, before averaging, the CW and interference score of the Stroop task z-scores, and the HSCT score z-scores were inverted as better performance was indicated with a higher score, whereas in the remaining assessments used, better performance was reflected by a lower score. Furthermore, the error-rates of the TEA and BDS performances were calculated and transformed).

**Table 3:** Decline in executive function abilities in older adults in comparison to young adults.

Task	*N*	Older adults z-scores	*t*	df	*p*-value	*d*
Dual-tasking						
TEA	20	0.03	0.15	42	0.885	0.05
PRP	22	0.04	0.28	44	0.782	0.08
Inhibition						
Stroop	25	1.27	5.14	49	**<0.001**	1.47
HSCT	23	0.78	2.69	47	**0.010**	0.78
Shifting						
Task switching task	21	0.74	2.75	45	**0.008**	0.82
TMT	19	−0.31	1.16	36	0.252	0.37
Updating						
BDS	25	0.08	0.26	51	0.794	0.07
N-back	23	0.53	1.99	47	0.053	0.58

The table contains the independent *t*-tests of the average outcome measures (z-scores) for all eight tasks between young and older adults.

BDS, Backward digit span task; HSCT, Hayling sentence completion task; PRP, Psychological Refractory Period paradigm; NB, N-back task; Stroop, Stroop task; TEA, Test for Everyday Attention; TMT, Trail making task. Bold *p*-values indicate significant effects (*p* < 0.05).

From each pair of task per EF, the task z-scores with the greatest decline among the four EF abilities due to healthy aging was observed to be the Stroop inhibition score, 1.27, followed by the task switching task shifting score, 0.78, the n-back updating, 0.53, and last the PRP DT, 0.04. Independent *t*-test analyses between the young and older adults z-scores showed significant differences in the Stroop, *p* < 0.001, HSCT, *p* = 0.010, and task switching task, *p* = 0.008, scores, suggesting a considerable difference between the groups’ performance outcomes. In order to determine which of these four EF measures presented with the most decline in the older adults and thus most sensitive to aging, a 2 × 4–factorial mixed ANOVAs with the factors Group (young adults vs. older adults) x 4 EF z-scores (dual-tasking vs. inhibition vs. shifting vs. updating) was performed. A main effect of Group*, F*(1, 41) = 20.47, *p* < 0.001, *Ƞ_p_^2^* = 0.33, main effect of EF z-score, *F*(3, 123) = 4.82, *p* = 0.003, *Ƞ_p_^2^* = 0.11, and an interaction, *F*(3, 123) = 4.93, *p* = 0.003, *Ƞ_p_^2^* = 0.11, were revealed. Six additional 2 × 2–factorial mixed ANOVAs with the factors Group (young adults vs. older adults) x EF z-score (between 2 of the EF) can be reviewed in Table 4.

**Table 4:** Age-related decline comparison between the four executive function abilities amongst the older adults.

Pairwise comparison	*N*		Inferential statistics		
	YA	OA	Main effect group	Main effect EF	Interaction Group × EF
Dual-tasking (PRP) vs. Inhibition (Stroop)	24	22	*F*(1, 44) = 19.97, ***p* < 0.001**, *Ƞ_p_*^2^ = 0.31	*F*(1, 44) = 13.98, ***p* = 0.001**, *Ƞ_p_*^2^ = 0.24	*F*(1, 44) = 18.24, ***p* < 0.001**, *Ƞ_p_*^2^ = 0.29
Dual-tasking (PRP) vs. Shifting (Task switching task)	23	20	*F*(1, 41) = 5.49, ***p* = 0.024**, *Ƞ_p_*^2^ = 0.12	*F*(1, 41) = 5.41, ***p* = 0.025**, *Ƞ_p_*^2^ = 0.12	*F*(1, 41) = 3.89, *p* = 0.055, *Ƞ_p_*^2^ = 0.09
Dual-tasking (PRP) vs. Updating (N-back)	24	20	*F*(1, 42) = 2.65, *p* = 0.111, *Ƞ_p_*^2^ = 0.06	*F*(1, 42) = 1.14, *p* = 0.292, *Ƞ_p_*^2^ = 0.03	*F*(1, 42) = 3.61, *p* = 0.064, *Ƞ_p_*^2^ = 0.08
Inhibition (Stroop) vs. Shifting (Task switching task)	26	23	*F*(1, 43) = 26.02, ***p* < 0.001**, *Ƞ_p_*^2^ = 0.38	*F*(1, 43) = 1.22, *p* = 0.275, *Ƞ_p_*^2^ = 0.03	*F*(1, 43) = 2.04, *p* = 0.160, *Ƞ_p_*^2^ = 0.05
Inhibition (Stroop) vs. Updating (N-back)	26	23	*F*(1, 47) = 5.68, ***p* = 0.021**, *Ƞ_p_*^2^ = 0.11	*F*(1, 47) = 5.68, ***p* = 0.021**, *Ƞ_p_*^2^ = 0.11	*F*(1, 47) = 20.57, ***p* < 0.001**, *Ƞ_p_*^2^ = 0.30
Shifting (Task switching task) vs. Updating (N-back)	24	21	*F*(1, 43) = 8.30, ***p* = 0.006**, *Ƞ_p_*^2^ = 0.16	*F*(1, 43) = 1.66, *p* = 0.205, *Ƞ_p_*^2^ = 0.04	*F*(1, 43) = 0.22, = 0.643, *Ƞ_p_*^2^ = 0.005

The four executive functions (average z-scores) with the largest decline rate were all compared with each other to assess the rate of decline between them using ANOVA analyses.

YA, Young Adults; OA, Old Adults; EF, Executive Function; N-back, N-back task; PRP, Psychological Refractory Period paradigm; Stroop, Stroop task. Bold *p*-values indicate significant effects (*p* < 0.05).

**Figure 1:**
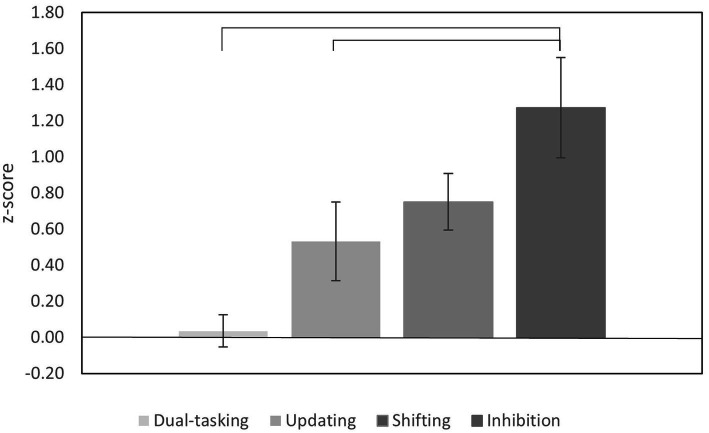
Decline in executive function abilities amongst the older adults. The bars represent the z-score data from the four executive function tasks presented in Table 4. Error bars represent the standard error of the mean. The bracket lines present the significant differences.

Of particular interest is the significance of the interaction because it reflects the difference in the aging effect between the two compared EFs. We found that inhibition, the strongest declining EF, showed significantly higher decline as compared to updating [interaction term, *F*(1, 47) = 20.57*, p* < 0.001, *Ƞ_p_^2^* = 0.30] and dual-tasking [*F*(1, 44) = 18.24, *p* < 0.001, *Ƞ_p_^2^* = 0.29], while the difference to shifting was not significant [*F*(1, 43) = 2.04, *p* = 0.160, *Ƞ_p_^2^* = 0.05]. Shifting, the EF with the second highest decline rate, showed higher rates of decline than dual-tasking, but this difference just failed to reach statistical significance [*F*(1, 41) = 3.89, *p* = 0.055, *Ƞ_p_^2^* = 0.09]. Shifting and updating did not differ significantly from each other [*F*(1, 43) = 0.22, *p* = 0.643, *Ƞ_p_^2^* = 0.005]. Finally, updating showed greater decline than dual-tasking, but again this difference just failed to reach significance [*F*(1, 42) = 3.61, *p* = 0.064, *Ƞ_p_^2^* = 0.08]. Taken together, the decline of all four EFs differed from each other (either statistically significant or marginally significant), except for updating and shifting which showed rather comparable decline rates (Figure 1).

#### Intercorrelation analysis

Correlation analyses were performed separately for young and older adults on the 8 average EF z-scores used in the previous section. Due to the multiple number of comparisons, i.e., 8 × 8, a Bonferroni corrected alpha of 0.05 / 64 = 0.00078125, was used to determine significance. There were no significant correlations in either age group (Tables 5, 6).

**Table 5:** Young adults correlation matrix of the average executive function *z*-score measures.

		TEA	PRP	HT	Stroop	TS	TMT	BDS
PRP	*r*	0.098						
	*p*	0.672						
	*N*	21						
HT	*r*	0.260	0.297					
	*p*	0.220	0.168					
	*N*	24	23					
Stroop	*r*	0.140	0.101	−0.096				
	*p*	0.534	0.640	0.657				
	N	22	24	24				
TS	*r*	0.103	0.349	0.287	−0.217			
	*p*	0.641	0.103	0.165	0.308			
	*N*	23	23	25	24			
TMT	*r*	0.032	0.480	0.007	0.425	0.199		
	*p*	0.908	0.051	0.978	0.070	0.444		
	*N*	16	17	17	19	17		
BDS	*r*	0.133	0.569	0.325	−0.114	0.109	0.161	
	*p*	0.537	0.004	0.105	0.578	0.597	0.511	
	*N*	24	24	26	26	26	19	
NB	*r*	0.332	0.232	0.100	0.191	0.119	0.064	0.486
	*p*	0.131	0.276	0.643	0.349	0.579	0.796	0.012
	*N*	22	24	24	26	24	19	26

*r*, Pearson’s correlation coefficient; *p*, *p*-value of *r*; BDS, Backward digit span task; HSCT, Hayling sentence completion task; PRP, Psychological Refractory Period paradigm; NB, n-back task; Stroop, Stroop task; TEA, Test for Everyday Attention; TMT, Trail making task; TS, Task switching task. Significance was determined with Bonferroni corrected alpha < 0.00078125, bold *p*-values.

**Table 6:** Older adults correlation matrix of the average executive function z-score measures.

		TEA	PRP	HT	Stroop	TS	TMT	BDS
PRP	*r*	−0.030						
	*p*	0.903						
	*N*	19						
HT	*r*	−0.275	0.545					
	*p*	0.254	0.011					
	*N*	19	21					
Stroop	*r*	0.581	−0.110	−0.115				
	*p*	0.007	0.625	0.602				
	*N*	20	22	23				
TS	*r*	0.499	−0.237	−0.284	0.132			
	*p*	0.035	0.314	0.212	0.569			
	*N*	18	20	21	21			
TMT	*r*	0.231	−0.074	−0.226	0.529	0.128		
	*p*	0.373	0.770	0.353	0.020	0.612		
	*N*	17	18	19	19	18		
BDS	*r*	0.397	0.072	−0.316	0.554	−0.037	0.482	
	*p*	0.083	0.751	0.141	0.004	0.872	0.037	
	*N*	20	22	23	25	21	19	
NB	*r*	0.311	0.482	0.000	0.253	0.359	0.142	0.165
	*p*	0.210	0.031	0.999	0.244	0.110	0.574	0.451
	*N*	18	20	22	23	21	18	23

*r*, Pearson’s correlation coefficient; *p*, *p*-value of *r*; BDS, Backward digit span task; HSCT, Hayling sentence completion task; PRP, Psychological Refractory Period paradigm; NB, n-back task; Stroop, Stroop task; TEA, Test for Everyday Attention; TMT, Trail making task; TS, Task switching task. Significance was determined with Bonferroni corrected alpha < 0.00078125, bold *p*-values.

Further analysis was performed on the outcome measures of all 8 tasks. The Bonferroni corrected alpha for these comparisons, i.e., 57 × 57, was 0.05 / 3,249 = 0.00001539, was used to determine significance. In the young adults, a total of 0.55% (9) significant positive cross correlations were observed, 0.43% (7) between the EFs dual-tasking (PRP task) and shifting (task switching task) measures, and 0.12% (2) between shifting (task switching task) and updating (n-back task) measures, see Supplementary Table S5. While in the older adults, a total of 0.18% (3) were seen, 0.06% (1) between the shifting task measures (TMT and the task switching task), and 0.12% (2) between shifting (task switching task) and updating (n-back task) measures, see Supplementary Table S6. Thus, correlations between the same EF task construct were only shown in the older adults.

## Discussion

This cross-sectional study assessed age decline on a range of tasks assessing the EFs dual-tasking, inhibition, shifting and updating between young and older adults. The results indicated that the older adults performed poorer in comparison to the young adults in several of the tasks, the PRP, Stroop, HSCT, task switching task, and n-back task. These findings are discussed in Supplementary material.

A further study aim was to compare the size of the age-related decline of these four EF abilities between the young and older adults. The analysis showed statistically significant differences between the rates with inhibition displaying the greatest decline, followed by shifting, updating, and dual-tasking. Additional cross-correlation analyses across these four EFs found no significant positive correlations, whilst analyses of the pairs of task measures showed an extremely small number of significant positive correlations, < 1%, in both age groups, following Bonferroni correction and the removal of correlations from the same task.

For the current comparison of the age-related decline across the EFs we averaged the performance measures of each task and picked the measure with the highest rate of decline from each of the four pairs of tasks. Significant differences between the decline rates showed inhibition to have the greatest level, then shifting, updating, and dual-tasking. As the first three EFs had much higher rates of decline in comparison to dual-tasking, it may suggest they may share a common underlying cognitive component (unity) as proposed by Miyake et al. (2000). While still being a heterogenous group of EFs (diversity). Dual-tasking did not load on this common factor, but as only one single DT paradigm was used, it was questioned whether the finding was task specific. Based on our findings, it would seem that dual-tasking ability is not as affected by the aging process as much as the other three EFs whereas decline in its ability has been shown to signal the onset of cognitive impairment (Baddeley et al., 1991; Lonie et al., 2009). This comparison of the rates of decline of these four EFs collectively has not been extensively researched in cognitive aging, or pathological conditions. Therefore, these novel findings are promising and may offer further contribution into understanding how these EFs are affected by aging. It is hoped that by recognizing a pattern of decline between these EFs, and possibly others, through consistent monitoring of decline rates in healthy individuals, deviations from it may signal the clinical presentation of a form of pathological cognitive impairment. Identifying early deficits is important as it facilitates early detection and possible treatment of a condition.

In a recent study, Saylik et al. (2022) reported in a functional magnetic resonance imaging (fMRI) study that all four EFs activated an overlapping set of brain areas (unity) particularly in the PFC, but also distinct areas (diversity). Thus, the different decline rates observed in this study could be linked with these unique regions activated during the specific EF processing which may also be linked to the central executive system (CES) of the WM model proposed by Baddeley and Hitch (1974). This system controls, coordinates, regulates, and integrates new information into and between the phonological loop (PL), visuospatial sketchpad (VSSP), and the episodic buffer slave systems (Baddeley, 2000). More specifically, as this singular system allocates cognitive resources in response to external information, these hypothetically independent systems may overlap in the processing of EFs, particularly of the WM domains, inhibition, shifting, and updating. While the diversity of EFs may relate to additional brain regions required for their processing such as the parietal cortex (Garavan et al., 1999; Sohn et al., 2000; Collette and Van der Linden, 2002; Roth and Courtney, 2007). So, it may be concluded that the diversity of EFs contributes to age-related cognitive decline. Thus, the findings of this study may have further our understanding of cognitive aging.

Further correlation analyses examined if measures from any of the four average EF measures or any of the task pair measures correlated. No positive significant correlations were found in comparing the average EF measures in either age group. With the individual task measures, very few correlations were observed in both groups following Bonferroni correction. With the young adults, correlations were observed between dual-tasking, PRP DT, and shifting, task switching task, measures, and between shifting, task switching task, and updating, n-back task, measures. Whereas the older adults showed correlations between EFs of the same construct, i.e., shifting, with the TMT and task switching task, and across different EFs, with the shifting, task switching task, and updating, n-back task measures. The dissimilar EF correlations could be attributed to the concept of unity amongst EFs, namely that inhibition, shifting, and updating, possess some common underlying factor as proposed by Miyake et al. (2000). Furthermore, the contrast in the correlations amongst the age groups might highlight an age effect, as reported by Glisky et al. (2020). Aging causes decrease in the efficiency of EF processing, i.e., the central executive system of the WM model proposed by Baddeley and Hitch (1974), resulting in the reallocation of limited cognitive processes to enhance performance. This is due to dedifferentiation and neural reorganization in the PFC resulting in EFs becoming less distinct and merging in older brains, causing reduction of selectivity of responses, resulting in more homogenous responses (Grady, 2012).

Nevertheless, The study presented must Be interpreted In consideration of a number of limitations. The sample sizes for both the young and older adult groups were relatively small, which introduces potential difficulties in generalizing the study results to the general population. In addition, the comparatively high level of cognitive reserve in the older participants may have contributed to the study findings by reducing the level of their cognitive decline, and hence resulting in better performance of some or all of the tasks (Meng and D’Arcy, 2012; Barulli et al., 2013; Cabeza et al., 2018). Moreover, this group of older individuals, and the university educated young adult participants were not a true representation of the general population. Resulting in an underestimation of cognitive decline. Another aspect is the numerous sites the assessments were conducted, i.e., participants’ homes, public areas, university grounds etc., particularly with the older adults, resulting in site variations that may have affected the assessment results.

Due to the explorative nature of the study, we had to calculate a number of tests for some analyses, e.g., when comparing all dependent variables and measures assessing DT performance, which increases the likelihood of type 1 errors. However, applying Bonferroni correction for this is rather strict and increases the likelihood of type 2 errors. Therefore, we decided to provide both findings, uncorrected and Bonferroni corrected *value of p*s. We based our interpretation of age-related declines on the original *value of p*s, as numerous previous studies have reported age-effects in identical or highly similar task paradigms and populations (McAlister and Schmitter-Edgecombe, 2016; Maldonado et al., 2020), which reduces the likelihood to commit type 1 errors. Furthermore, showing age-related decline for each individual EF as such was not central to this paper, as this has been shown previously. Instead, the comparison of the magnitude of the age-related decline across the four EFs was the most relevant aspect. Importantly, this comparison of effect sizes across the EFs is not dependent on whether the individual effects significantly differ from zero or not.

Lastly, the moderate level of depression in the older adult group may have negatively impacted their EF performance (Rock et al., 2014; Wang et al., 2017), although clinical depression and not general depression has been stated to negatively affect various EFs differently (James et al., 2021). For instance, DT seems to be unaffected (Kaschel et al., 2009; Foley et al., 2011, 2013). Whilst it has also been reported that high levels of education may offset the effect of depression (Chlapecka et al., 2020; James et al., 2021) and therefore may not affect cognitive performance greatly in such individuals. Nonetheless, no individual recruited into the study had been diagnosed clinically with depression. Furthermore, as these older adults presented with a comparable normal cognitive status with the young adults as shown with the MMSE and MoCA scores, it is conceivable that depression did not adversely affect their performance. However, it may also be that some of these individuals might be depressed and the age-related decline in EF ability observed could in fact be due to depression or may have added to the findings.

Despite these limitations, a strength of the study was the employment of multiple tasks in the assessment of each of the four cognitive EF domains, which provided a unique assessment of the difference in task measures. Moreover, the age-related EF decline findings are consistent with results reported from previous EF studies performed in young and older adults (Braver and West, 2008; Maldonado et al., 2020) thus validating this study. Still future studies are required, such as a longitudinal study with the inclusion of middle-aged and very old adults together with those living with pathological impairment, i.e., mild cognitively impairment and/or Alzheimer’s disease to allow for a comprehensive review of EF decline. This would allow for further insight into the decline rate of the four EFs in these neurodegenerative conditions and thus, the tracking and comparison of decline of individual EFs by detecting deviations from ‘normal’ decline rates. Such research could have profound clinical implications by allowing for the identification of potential MCI and/or dementia patients who might benefit from early cognitive training. Also, separating and researching the performance of low and highly educated participants across the groups would be advantageous to gain additional knowledge on the concept of cognitive reserve, cognitive aging, and possible pathological impairment.

Additionally, investigating a vaster array of EFs, including visuospatial and planning abilities for a thorough understanding of decline of cognitive abilities should be conducted, as EFs coordinate and collaborate with each other to bring about an action. Similarly, increasing the number of tasks used to assess each EF may be implemented, as shown with the findings of this study different outcomes may be seen when assessing the same group of participants. This will further assess the concept of the unity and diversity of EFs. Tasks including the Tower of London (Shallice, 1982), or Tower of Hanoi (Humes et al., 1997), or the Six Elements test (Wilson et al., 1996) may be employed for such assessments. Moreover, manipulating and comparing of the stimuli used in the PRP, task switching, and n-back tasks, should be considered. For example, the use of picture/symbol, letter, or word stimuli can be explored to determine what effect is made on performance outcomes, in comparison to what was observed with the use of digits. Particularly in older adults, as they have been reported to perform better with lexical stimuli in comparison to non-lexicon stimuli (Balota, 1996). Although EFs are thought to be stimulus independent, research has shown these various types of stimuli use different brain networks for their processing, hence task performance may differ depending on the extent at which such networks are affected by brain aging (Seifert, 1997; Azizian et al., 2006; Kahlaoui et al., 2007; Carreiras et al., 2015a, 2015b). Thus, further assessment of the EFs using different tasks to those employed in this study and with various stimuli would be beneficial.

## Conclusion

First, the present cross-sectional study demonstrated that the four EFs inhibition, shifting, updating, and dual-tasking show decline with healthy aging. Second, each EF was assessed with a pair of two different tasks, and we found surprisingly little correlations among the pairs of tasks aiming to measure the same EF, highlighting the importance of task choice when investigating EFs. Finally, and most importantly, we compared the relative size of the decline across the four EFs, in a within-subject design. We found significant differences in the decline, with inhibition showing the greatest and dual-tasking the smallest decline. Therefore, our findings suggest that speaking of “age-related decline in executive functions” may be too unspecific, and a more detailed characterisation of the exact executive functions would be beneficial. Future studies should expand this line of research to pathological aging to test whether the differential rates of decline may prove as a useful tool for differential diagnosis of dementias and other conditions.

## Data availability statement

The raw data supporting the conclusions of this article can be downloaded under https://doi.org/10.17633/rd.brunel.21937595.

## Ethics statement

The studies involving human participants were reviewed and approved by Brunel University’s Life Sciences Ethics Committee. The patients/participants provided their written informed consent to participate in this study.

## Author contributions

MI oversaw all aspects of the current study and wrote the manuscript. AS designed the computerized EF tasks and helped with manuscript preparation. All authors designed the manuscript. All authors read and approved the final manuscript.

## Conflict of interest

The authors declare that the research was conducted in the absence of any commercial or financial relationships that could be construed as a potential conflict of interest.

## Publisher’s note

All claims expressed in this article are solely those of the authors and do not necessarily represent those of their affiliated organizations, or those of the publisher, the editors and the reviewers. Any product that may be evaluated in this article, or claim that may be made by its manufacturer, is not guaranteed or endorsed by the publisher.

## Footnotes

^1^ www.neurobs.com
