# Supplementary material for: Executive function abilities in cognitively healthy young and older adults—A cross-sectional study
Source: Front Aging Neurosci. 2023 Feb 8;15:976915. doi: 10.3389/fnagi.2023.976915 (PMC9945216; doi:10.3389/fnagi.2023.976915)
Supplement: Supplementary file 1 [file Data_Sheet_1.zip › SuppMaterial.DOCX]

Supplementary Material

Supplementary Information for

**Executive Function Abilities in Cognitively Healthy Young and Older Adults, A Cross-Sectional Study**

**Dr Mojitola I Idowu, Dr Andre J Szameitat**

Corresponding Author: Dr Mojitola I Idowu

Email: Mojitola.Idowu@outlook.com

**This file includes:**

**Results**

**Discussion**

**References**

**Tables S1 to S4.**

**RESULTS**

***Dual-tasking***

In the PRP task, to test whether there was significant age-related decline in the performance between the ST and DT conditions at SOA 0ms where participants were required to respond to RT1 before RT2, four 2 × 2- factorial mixed ANOVAs with the factors Group (young adults vs older adults) and task (ST vs DT) were calculated separately for RT1-auditory and RT2-visual, RTs and error-rates. However, the analysis for RT2 should show the largest effects (Szameitat & Students, 2022) as the costs for RT are a sub-set of the RT2 costs.

The RT cost analysis for the RT2 showed no main effect of Group, *F*(1, 44) = 2.54, *p* = 0.118, *Ƞ_p_^2^* = 0.06, but a main effect of the task condition, *F*(1, 44) = 288.85, *p* < 0.001, *Ƞ_p_^2^* = 0.87, where RT was longer for the DT condition. There was no interaction, *F*(1, 44) = 0.90, *p* = 0.347, *Ƞ_p_^2^* = 0.02 as both groups performed comparable. For the error-rate cost, a main effect of Group was revealed, *F*(1, 44) = 12.43, *p* = 0.001, *Ƞ_p_^2^* = 0.22, with the older adults performing more accurately, and a main effect of the PRP task condition, *F*(1, 44) = 21.28, *p* < 0.001, *Ƞ_p_^2^* = 0.33, as DT resulted in an increased proportion of errors. No interaction, *F*(1, 44) = 3.94, *p* = 0.054, *Ƞ_p_^2^* = 0.08, was found. Thus, the groups performed similarly.

For the RT1, the RT cost analysis found no main effect of the Group, *F*(1, 44) = 0.19, *p* = 0.662, *Ƞ_p_^2^* = 0.004. A main effect of the task condition was found, *F*(1, 44) = 111.96, *p* < 0.001, *Ƞ_p_^2^* = 0.72, as DT resulted in longer RTs in both groups. There was no interaction *F*(1, 44) = 0.07, *p* = 0.790, *Ƞ_p_^2^* = 0.002. With the mean error-rate cost, there was a main effect of Group, *F*(1, 44) = 6.79, *p* = 0.012 (not significant following Bonferroni correction, alpha of 0.003), *Ƞ_p_^2^* = 0.13, and main effect of the task condition, *F*(1, 44) = 6.94, *p* = 0.012 (not significant following Bonferroni correction), *Ƞ_p_^2^* = 0.14, due to the older adults producing less errors in both task conditions, as well as both groups producing more errors during the DT. However, the interaction was not significant, *F*(1, 44) = 2.33, *p* = 0.134, *Ƞ_p_^2^* = 0.05.

***Inhibition***

Although the analysis of the RT inhibition costs was significantly different between the age groups, additional analysis of the HSCT categorized scaled scores only showed significance with the part 2 score, *t*(47) = 2.47, *p* = 0.017 (not significant following Bonferroni correction, alpha of 0.005), *d* = 0.72. There was no difference between the categorization of the part 1 score, *t*(47) = -1.64, *p* = 0.107, *d* = 0.48, or the error-rate performance in part 2, *t*(47) = 0.84, *p* = 0.404, *d* = 0.25.

With the second inhibitory task, the Stroop task, a 2 x 3-factorial mixed ANOVA with the factors Group (young adults vs older adults) x Stroop section (C vs W vs CW), was calculated to assess performance differences between the groups and task section. Results found a main effect of Group, *F*(1, 49) = 9.74, *p* = 0.003, *Ƞ_p_^2^* = 0.17, as the younger group were better performers. There was a main effect of the Stroop section, *F*(2, 98) = 412.56, *p* < 0.001, *Ƞ_p_^2^* = 0.89, indicating differences in the performance of these sections. The group difference was qualified by an interaction effect, *F*(2, 98) = 5.67, *p* = 0.005, *Ƞ_p_^2^* = 0.10, showing that the age-related cognitive decline was different for the different sections. Further analysis was therefore conducted to compare performance between each repetition section, and between each repetition section and the inhibition section by calculating three 2 x 2–factorial mixed ANOVAs, with the factors Group (young adults vs older adults) x Stroop section (C vs W, C vs CW, W vs CW). Results for “C vs W” analysis found no main effect of Group, *F*(1, 49) = 2.43, *p* = 0.125, *Ƞ_p_^2^* = 0.05, a main effect of the Stroop section, *F*(1, 49) = 151.26 , *p* < 0.001, *Ƞ_p_^2^* = 0.76, as the W section was performed with more accuracy than the C section. However, no interaction effect was observed, *F*(1, 49) = 0.69, *p* = 0.409, *Ƞ_p_^2^* = 0.01, signifying that although the sections were completed differently there was no difference between the groups’ performance.

Analysis between “C vs CW” showed a main effect of Group, *F*(1, 49) = 13.87, *p* = 0001, *Ƞ_p_^2^* = 0.22, a main effect of the Stroop section, *F*(1, 49) = 294.85, *p* < 0.001, *Ƞ_p_^2^* = 0.86, indicating the accuracy performance of the sections were different, and an interaction, *F*(1, 49) = 6.65, *p* = 0.013 (not significant following Bonferroni correction), *Ƞ_p_^2^* = 0.12. The “W vs CW” analysis found a main effect of Group, *F*(1, 49) = 12.19, *p* = 0.001, *Ƞ_p_^2^* = 0.20, a main effect of the Stroop section, *F*(1, 49) = 773.10, *p* < 0.001, *Ƞ_p_^2^* = 0.94, suggesting a difference in performance, and an interaction, *F*(1, 49) = 9.94, *p* = 0.003, *Ƞ_p_^2^* = 0.17. Therefore, indicating both groups completed the sections with different accuracy. These results suggest performance of the two repetition sections, C and W, were less demanding than the inhibition section, CW, due to these being non-conflict sections.

A 2 × 2- factorial mixed ANOVA comparing the inhibitory costs with the factors Group (young adults vs older adults) and conditions (CW-C, CW-W), showed a main effect of Group, *F*(1, 49) = 11.53, *p* = 0.001, *Ƞ_p_^2^* = 0.19, and a main effect of cost type, *F*(1, 49) = 151.26, *p* < 0.001, *Ƞ_p_^2^* = 0.76. No interaction was found, *F*(1, 49) = 0.69, *p* = 0.409, *Ƞ_p_^2^* = 0.01, thus there was no difference between the groups’ costs. Both groups possessed lower (CW-W) costs than CW-C costs, indicating a difference between the C and W performances.

***Updating***

To assess the RT and error-rate results of all the n-back conditions between the groups, two 2 × 4–factorial mixed ANOVAs with the factors Group (young adults vs older adults) x n-back condition (0- vs 1- vs 2- vs 3-back) were conducted. Results for the RTs showed a main effect of Group, *F*(1, 47) = 33.27, *p* < 0.001, *Ƞ_p_^2^* = 0.41, as the groups performed differently but no main effect of the task condition, *F*(3, 141) = 1.34, *p* = 0.263, *Ƞ_p_^2^* = 0.03. The increase in n-back position did not affect the time taken for the participants to complete the trial blocks. This translated as no age-related difference in the RTs as there was no interaction effect, *F*(3, 141) = 0.07, *p* = 0.978, *Ƞ_p_^2^* = 0.001. Both groups performed comparably. However, the error-rate results showed a main effect of Group, *F*(1, 47) = 17.33, *p* < 0.001, *Ƞ_p_^2^* = 0.27, a main effect of the task condition, *F*(3, 141) = 268.68, *p* < 0.001, *Ƞ_p_^2^* = 0.85, demonstrating the classic effect of this task with an increase in errors produced as the n-back position increased, and an interaction effect, *F*(3, 141) = 6.66, *p* < 0.001, *Ƞ_p_^2^* = 0.12. The increase in task position caused an increase in task difficulty which affected the older adults substantially more than the young adults resulting in a reduction in accuracy performance.

Pairwise analysis between each n-back condition was compared between the groups. At 0-back, significance was only found in the mean RT, *t*(48) = -6.84, *p* < 0.001, *d* = 1.97, [error-rates, *t*(48) = -1.39, *p* = 0.171, *d* = 0.40] signifying that the older adults spent longer in completing the condition but produced comparable errors with the younger adults, in the least demanding condition of the task. Significant differences were found between the groups during the remaining conditions in the mean RTs [1-back, *t*(48) = -4.53, *p* < 0.001, *d* = 1.31; 2-back, *t*(48) = -4.14, *p* < 0.001, *d* = 1.20; 3-back, *t*(47) = -3.77, *p* < 0.001, *d* = 1.10], and error-rates [1-back, *t*(48) = -3.46, *p* = 0.001, *d* = 1.00; 2-back, *t*(48) = -3.61, *p* = 0.001, *d* = 1.04; 3-back, *t*(47) = -4.18, *p* < 0.001, *d* = 1.22]. Therefore, signifying that the older adults possessed higher RTs and generated more errors during these conditions.

To further examine the groups’ performances, a series of pairwise comparisons for the different conditions, i.e., 0 vs 1, 0 vs 2, 1 vs 3 etc., between the groups’ RTs and error-rates, was conducted. Following Bonferroni correction, all the RT comparisons were observed as being statistically insignificant, all *t*(49) = -2.43, *p* < 0.019; all *p* > 0.00833 (0.000833 following Bonferroni correction), implying the groups had approximately the same mean RTs between all the pairwise comparisons. However, significant differences in the error-rates between all the n-back conditions in each group, and between the groups was demonstrated, e.g., *t*(49) = -7.99, *p* < 0.001; all *p* < 0.001.

# DISCUSSION

Aging was observed to have affected DT performance in the RTs of the PRP effect with the PRP paradigm (Pashler, 1984; Welford, 1952). The older group produced a higher mean RT difference between performance in the SOA 0 ms and 1000 ms, which has similarly been reported by a number of researchers in cross-sectional age studies (Allen et al., 1998; Glass et al., 2000; Hartley, 2001; Hartley et al., 1999; Hein & Schubert, 2004; Verhaeghen et al., 2003). In the PRP paradigm, a key assumption is that at short SOAs strong interference arises because both tasks compete for a central attentional bottleneck, and that this interference is greatly reduced at longer SOAs. The older participants were slowed down in the short SOA condition significantly more than the young participants, suggesting the older participants’ abilities to coordinate task processing at the central bottleneck has declined (Greenwald, 1972; Greenwald & Shulman, 1973; Hartley et al., 2015; Maquestiaux et al., 2008).

With respect to the age-related decline, Allen et al. (1998) found that older adults differed in their strategy to do the task by withholding the response for task one until the response of task two was ready (also known as response grouping). Further studies where the task stimulus and the response characteristics have been manipulated have proposed other reasons for the nature of the age effects. These have included process-specific slowing, the increased employment of cautious task-coordination strategies (Glass et al., 2000), response-generation processes (Hartley, 2001), and input processes (Hein & Schubert, 2004). Generalized slowness has also been suggested, however, after controlling for this Hartley et al. (1999) still observed age effects when manual responses were required for both tasks. Nevertheless, this was concluded to be due to the use of the response type and slowness has still been proposed (Glass et al., 2000) as older adults are thought to be approximately 1.5 times slower than younger adults in general (Maquestiaux, 2016). Accordingly, it is unknown which of these processes caused the substantial PRP effect in RT of the older adults assessed in this study.

Regarding the TEA DT telephone code search subtest (Robertson et al., 1994), the similar performance between the ages may stem from the task being insensitive to age-associated performance deficits in multitasking, as well as the small number of participants recruited in the groups. However, it may simply imply that the older adults successfully engaged in a compensatory strategy as described by the strategy-deficit hypothesis (Bailey et al., 2009).

Age-related decline in inhibitory control was observed in the interference score of the Stroop task (Golden, 1978), and in the inhibition RT cost (RT difference between incongruent and congruent sections) of the HSCT (Burgess & Shallice, 1997), findings that have been well supported in previous research (Albinet et al., 2012; Bielak et al., 2006; Borella et al., 2008, 2009, 2011; Bruyer et al., 1995; Bugg et al., 2007; Cervera-Crespo & González-Alvarez, 2017; Graf et al., 1995; Houx et al., 1993; Mayas et al., 2012; McCabe et al., 2005; Morrone et al., 2010; Pettigrew & Martin, 2014; Spieler et al., 1996; Tournier et al., 2014; Troyer et al., 2006; Uttl & Graf, 1997; Zimmermann et al., 2017). The results are also consistent with the inhibition deficit theory (Hasher & Zacks, 1988) in that older adults are less capable of suppressing or ignoring irrelevant information in comparison to young adults, highlighted in a meta-analysis by Rey-Mermet & Gade (2018).

With the Stroop task, older adults are said to be affected more by the increased interference of the word processing during the incongruent section which requires time to resolve the conflict (Spieler et al., 1996; Uttl & Graf, 1997). However, the age-related decline in the interference score is not only due to this cognitive slowing but also to other cognitive changes, including decreased response inhibition (Bugg et al., 2007; Mayas et al., 2012; Spieler et al., 1996). Whereas when completing the high-cloze sentences in the incongruent part 2 of the HSCT, performance is dependent on the ability to refrain from producing a predominant word, and instead to produce an irrelevant word (Martin et al., 2019). In particular for the production of an incongruent word, fluid intelligence in the form of semantics and contextual meaning is required, and age-associated decline in fluid intelligence has been proposed to account for some of the age effects seen in the HSCT RTs (Bielak et al., 2006; Zimmermann et al., 2017). Therefore, the HSCT may be sensitive to both decline in inhibition and/or in fluid intelligence.

Moreover, although both these tasks claim to assess inhibitory abilities, multiple different inhibitory processes have been proposed (Borella et al., 2009; Nigg, 2000). For example, the Stroop task has been suggested to measure inhibition of automatic stimulus-response associations at the response level, and the Stroop interference score is understood to assess the ability to inhibit the disruptiveness of the stimulus (Borella et al., 2009; Martin et al., 2019). While the HSCT measures inhibition on the semantics/contextual level, where participants consciously suppress words to sentences they are accustomed to from entering WM (Borella et al., 2009; Martin et al., 2019). The data therefore seem to be in line with these theories and it can be concluded that potentially different forms of inhibition are affected by increasing age, as both tests observed decline. Thus, highlighting one of the issues that may be encountered with EF tasks, i.e., the assessment of a different subtypes of the same EF, e.g., response inhibition, cognitive inhibition (Diamond, 2013). Despite this, these were the only tasks to both show age-related performance deficits for the same EF. Thus, it seems the inhibition deficit hypothesis (Hasher & Zacks, 1988; Lustig et al., 2007) may be accurate in stating impairment in inhibition is the primary source of age-related deficits reported in the performance of numerous cognitive tasks, especially those involving WM (Campbell et al., 2020; Hasher et al., 2008; Koch et al., 2010; Persad et al., 2002). Also, Gilsoul et al. (2019) found inhibition partly mediated the effect of aging of the three other EFs.

With the task switching task (Rogers & Monsell, 1995), age-related decline in shifting ability was evident with the local- and global-shift RT costs, and the error-rate mixing-cost. Such age effects is consistent with the findings of many studies with this test (Brandt & Benedict, 2001; Hirsch et al., 2016; Kramer et al., 1999; Kray et al., 2002; Kray & Lindenberger, 2000; Meiran et al., 2001; Moretti et al., 2018; Reimers & Maylor, 2005; Wasylyshyn et al., 2011).

The three shift types differ in the involved cognitive processes reflecting the maintenance of multiple task configurations in WM, particularly during the pseudo-random presentation of the shifting task with two response sets in one trial block in comparison to one in each of the two repetition trial blocks (Huff et al., 2015; Wasylyshyn et al., 2011). The global shift costs comprise of the processes employed during both local shift cost and mixing cost performances by assessing the difference between the shifting trial of the mixed block and the repetition trials of the pure blocks. It is understood to measure the set-up cost associated with the maintenance and scheduling of the two mental task sets, in addition to the load on WM (Kray & Lindenberger, 2000; Li et al., 2019; Mayr, 2001). Whilst local shift costs examine performance between shift and repetition trials within mixed blocks and thus the demand to shift focus from task to task immediately. This translates to the process of removing information from the previous task ‘unloading’ (e.g., in response to a cue), before completing information for the new task-set ‘uploading’. However, the unloading process is not one hundred percent, and the memory of the previous task may remain, until the target is presented, then the shift is completely finalized i.e., task-set inertia (Monsell, 2003). The mixing-costs compare performance of the repetition trials in the mixed block and the pure blocks, which, as mentioned earlier, identifies the increased demands of retaining two task sets in short-term memory. Therefore, these costs show the different task demands for maintaining and selecting between the two possible response sets (Reimers & Maylor, 2005).

Regarding the observed age effects, it has been proposed that older adults are unable to efficiently maintain and coordinate two tasks in WM (Bopp & Verhaeghen, 2009; Gajewski, Ferdinand, et al., 2018; Kray et al., 2002; Kray & Lindenberger, 2000; Wasylyshyn et al., 2011). The decline in performance is thought to be the result of general slowing (Van Asselen & Ridderinkhof, 2000) as theorized in the processing-speed theory (Salthouse, 1996). This may account for the global shift and local shift RT costs as the time taken to prepare for task set, e.g., in response to a task cue (Hirsch et al., 2016; Monsell, 2003), would be affected. Furthermore, deficit in local shift costs may also be associated with the inhibition deficit theory (Hasher & Zacks, 1988) due the inefficiency of this population to inhibit the old, to-be-switched-away-from, task (Kray & Lindenberger, 2000; Velichkovsky et al., 2020). Nevertheless, age effects of global shift error-rate cost have been reported as well as RT cost which may be related to the stimulus types employed (Huff et al., 2015; Velichkovsky et al., 2020). Furthermore, in a meta-analysis by Wasylyshyn et al. (2011), it was reported that local shift costs where not age sensitive while global shift costs were. Whereas, the error-rate mixing-cost might reflect reduction in executive control of completing multiple task conditions (Braver et al., 2003; Chang et al., 2020) in addition to global slowing (Van Asselen & Ridderinkhof, 2000). Thus, age effects in the RT mixing-cost have also been reported (Eich et al., 2018; Meiran et al., 2001). Additionally, it has been proposed that significant differences in cost types can be caused by different WM demands, which can be reduced by the use of task cues (Gajewski, Ferdinand, et al., 2018; Rubinstein et al., 2001), as were utilized in this study. In line with this, age-associated decline in shifting ability is said to increase with task uncertainty, as with the removal of environmental prompts to guide behavior in daily life (Kray et al., 2002; Moretti et al., 2018).

No age-related decline in performance with the utilization of the TMT (Reitan, 1992) was observed. This insignificant finding has also been reported by researchers including Ebert & Anderson (2009), Maquestiaux et al. (2010) and Wecker et al. (2000). Though numerous others have described age-related decline (Hamdan & Hamdan, 2009; Tombaugh, 2004; Wecker et al., 2005; Woods et al., 2015; Zimmermann et al., 2017). This discrepancy particularly in part B of the task is thought to be related to the intelligence and/or education level of the older group (Boll & Reitan, 1973; Hashimoto et al., 2006; Hester et al., 2005; Kennedy, 1981; Rasmusson et al., 1998; Tombaugh, 2004), as it has been shown better educated, intelligent individuals show less cognitive decline which may have occurred in this study.

Age-related differences in WM updating capacity was seen with the employment of the spatial n-back task (Kirchner, 1958). The task is largely dependent on WM where greater task difficulty, i.e., higher *n*, is linked with the occurrence of an updating deficit. In more detail, a significantly larger error-rate cost was found in the older group, a result which has been previously reported although RT costs have also been shown (Bopp & Verhaeghen, 2018). It is thought to be due to deficits regarding the focus of attention within WM (Bopp & Verhaeghen, 2018). N-back positions over 1-back increases WM demands, causing age-related deficits in the organizing and managing of new incoming information, i.e., the encoding, storage, and processing of items not in the focus of attention, suggesting an issue with focus switching processes and decline in attention (Bopp & Verhaeghen, 2018; Kirchner, 1958). The fact that we observed age-related costs in accuracy only has been theorized to be an age-related decrease in WM storage or availability (Vaughan et al., 2008; Verhaeghen & Basak, 2005). Speed is understood to be linked with the accessibility and efficiency of retrieval processes in WM (Linares et al., 2016; Vaughan et al., 2008; Verhaeghen & Basak, 2005). In turn, the performance deficits observed in older adults are proposed to be due to a decrease in the efficiency to maintain cognitive control, especially during high demand task situations. This is said to be the consequence of structural changes in the prefrontal cortex as described by the executive attention framework by Engle (2002) and Engle & Kane (2004).

The lack of age-related performance impairments with the BDS task might also be due to the level of education of the older group as with the TMT performance, as well-educated individuals have been shown to show better performance in this task (Han et al., 2014; Ostrosky‐Solís & Lozano, 2006; Woods et al., 2011). Interestingly, there is evidence that aging negatively affects spatial WM task performance greater than verbal WM task performance (Hale et al., 2011), which could account for the results seen here. Additionally, n-back task performance across the age groups has been reported to use different functions (Gajewski, Hanisch, et al., 2018). Young adults are alleged to use EFs such as interference control, shifting and updating. Whilst older adults are believed to rely more on attention, and to a smaller degree, shifting and updating (Gajewski, Hanisch, et al., 2018), indicating a change in processing strategy between the ages.

**REFERENCES**

Albinet, C. T., Boucard, G., Bouquet, C. A., & Audiffren, M. (2012). Processing speed and executive functions in cognitive aging: How to disentangle their mutual relationship? *Brain and Cognition*, *79*(1), 1–11. https://doi.org/10.1016/j.bandc.2012.02.001

Allen, P. A., Smith, A. F., Vires-Collins, H., & Sperry, S. (1998). The psychological refractory period: Evidence for age differences in attentional time-sharing. *Psychology and Aging*, *13*(2), 218–229. https://doi.org/10.1037/0882-7974.13.2.218

Bailey, H., Dunlosky, J., & Hertzog, C. (2009). Does Differential Strategy Use Account for Age-Related Deficits in Working-Memory Performance? *Psychology and Aging*, *24*(1), 82–92. https://doi.org/10.1037/a0014078

Bielak, A. A. M., Mansueti, L., Strauss, E., & Dixon, R. A. (2006). Performance on the Hayling and Brixton tests in older adults: Norms and correlates. *Archives of Clinical Neuropsychology*, *21*(2), 141–149. https://doi.org/10.1016/j.acn.2005.08.006

Boll, T. J., & Reitan, R. . (1973). Effect of age on performance of the Trail Making Test. *Perceptual and Motor Skills*, *36*(3), 691–694.

Bopp, K. L., & Verhaeghen, P. (2009). Working Memory and Aging: Separating the Effects of Content and Context. *Psychology and Aging*, *24*(4), 968–980. https://doi.org/10.1037/a0017731

Bopp, K. L., & Verhaeghen, P. (2018). Aging and n-Back Performance: A Meta-Analysis. *The Journals of Gerontology Series B: Psychological Sciences and Social Sciences*, *00*(February 2018), 1–12. https://doi.org/10.1093/geronb/gby024

Borella, E., Carretti, B., & De Beni, R. (2008). Working memory and inhibition across the adult life-span. *Acta Psychologica*, *128*(1), 33–44. https://doi.org/10.1016/j.actpsy.2007.09.008

Borella, E., Delaloye, C., Lecerf, T., Renaud, O., & De Ribaupierre, A. (2009). Do age differences between young and older adults in inhibitory tasks depend on the degree of activation of information? *European Journal of Cognitive Psychology*, *21*(2–3), 445–472. https://doi.org/10.1080/09541440802613997

Borella, E., Ludwig, C., Fagot, D., & De Ribaupierre, A. (2011). The effect of age and individual differences in attentional control: A sample case using the Hayling test. *Archives of Gerontology and Geriatrics*, *53*(1). https://doi.org/10.1016/j.archger.2010.11.005

Brandt, J., & Benedict, R. (2001). *The Hopkins Verbal Learning Test – Revised*. 1–7.

Braver, T. S., Reynolds, J. R., & Donaldson, D. I. (2003). Neural mechanisms of transient and sustained cognitive control during task switching. *Neuron*, *39*(4), 713–726. https://doi.org/10.1016/S0896-6273(03)00466-5

Bruyer, R., Linden, M. Van der, Rectem, D., & Galvez., C. (1995). Effects of age and education on the Stroop interference. *Archives de Psychologie*, *63*, 257–267.

Bugg, J. M., DeLosh, E. L., Davalos, D. B., & Davis, H. P. (2007). Age differences in stroop interference: Contributions of general slowing and task-specific deficits. *Aging, Neuropsychology, and Cognition*, *14*(2), 155–167. https://doi.org/10.1080/138255891007065

Burgess, P. W., & Shallice, T. (1997). The Hayling and Brixton Tests. In *Bury St Edmunds: Thames Valley Test Company.*

Campbell, K. L., Lustig, C., & Hasher, L. (2020). Aging and inhibition: Introduction to the special issue. *Psychology and Aging*, *35*(5), 605–613. https://doi.org/10.1037/pag0000564

Cervera-Crespo, T., & González-Alvarez, J. (2017). Age and semantic inhibition measured by the hayling task: A meta-analysis. *Archives of Clinical Neuropsychology*, *32*(2), 198–214. https://doi.org/10.1093/arclin/acw088

Chang, W. P., Shen, I. H., Wen, C. P., & Chen, C. L. (2020). Age-Related Differences Between Young and Old Adults: Effects of Advance Information on Task Switching. *Perceptual and Motor Skills*, *127*(6), 985–1014. https://doi.org/10.1177/0031512520930872

Diamond, A. (2013). Executive Functions. *Annual Review of Psychology*, *64*, 135–168. https://doi.org/10.1146/annurev-psych-113011-143750

Ebert, P. L., & Anderson, N. D. (2009). Proactive and retroactive interference in young adults, healthy older adults, and older adults with amnestic mild cognitive impairment. *Journal of the International Neuropsychological Society*, *15*(1), 83–93. https://doi.org/10.1017/S1355617708090115

Eich, T. S., MacKay-Brandt, A., Stern, Y., & Gopher, D. (2018). Age-Based Differences in Task Switching Are Moderated by Executive Control Demands. *The Journals of Gerontology Series B: Psychological Sciences and Social Sciences*, *73*(6), 954–963. https://doi.org/10.1093/geronb/gbw117

Engle, R. W. (2002). Working memory capacity as executive attention. *Current Directions in Psychological Science*, *11*(1), 19–23.

Engle, R. W., & Kane, M. J. (2004). Executive Attention, Working Memory Capacity, and a Two-Factor Theory of Cognitive Control. *Psychology of Learning and Motivation - Advances in Research and Theory*, *44*, 145–199. https://doi.org/10.1016/S0079-7421(03)44005-X

Gajewski, P. D., Ferdinand, N. K., Kray, J., & Falkenstein, M. (2018). Understanding sources of adult age differences in task switching: Evidence from behavioral and ERP studies. *Neuroscience and Biobehavioral Reviews*, *92*(June 2017), 255–275. https://doi.org/10.1016/j.neubiorev.2018.05.029

Gajewski, P. D., Hanisch, E., Falkenstein, M., Thönes, S., & Wascher, E. (2018). What does the n-Back task measure as we get older? Relations between working-memory measures and other cognitive functions across the lifespan. *Frontiers in Psychology*, *9*(NOV), 1–17. https://doi.org/10.3389/fpsyg.2018.02208

Gilsoul, J., Simon, J., Hogge, M., & Collette, F. (2019). Do attentional capacities and processing speed mediate the effect of age on executive functioning? *Aging, Neuropsychology, and Cognition*, *26*(2), 282–317. https://doi.org/10.1080/13825585.2018.1432746

Glass, J. M., Schumacher, E. H., Lauber, E. J., Zurbriggen, E. L., Gmeindl, L., Kieras, D. E., & Meyer, D. E. (2000). Aging and the Psychological Refractory Period: Task Coordination Strategies in Young and Old Adults. *Psychology and Aging*, *15*(4), 571–595. https://doi.org/10.1037/0882-7974.15.4.571

Golden, C. J. (1978). *A manual for the clinical and experimental use of the Stroop color and word test*.

Graf, P., Uttl, B., & Tuokko, H. (1995). Color- and Picture-Word Stroop Tests: Performance Changes in Old Age. *Journal of Clinical and Experimental Neuropsychology*, *17*(3), 390–415. https://doi.org/10.1080/01688639508405132

Greenwald, A. G. (1972). On doing two things at once: Time sharing as a function of ideomotor compatibility. *Experimental Psychology*, *94*(1), 52–57.

Greenwald, A. G., & Shulman, H. G. (1973). On doing two things at once. II. Elimination of the psychological refractory period effect. *Journal of Experimental Psychology*, *101*(1), 70–76.

Hale, S., Rose, N. S., Myerson, J., Strube, M. J., Sommers, M., Tye-Murray, N., & Spehar, B. (2011). The Structure of Working Memory Abilities Across the Adult Life Span. *Psychology and Aging*, *26*(1), 92–110. https://doi.org/10.1037/a0021483

Hamdan, A. C., & Hamdan, E. M. L. R. (2009). Effects of age and education level on the Trail Making Test in a healthy Brazilian sample. *Psychology & Neuroscience*, *2*(2), 199–203. https://doi.org/10.3922/j.psns.2009.2.012

Han, J. Y., Seo, E. H., Yi, D., Sohn, B. K., Choe, Y. M., Byun, M. S., Choi, H. J., Kim, S. G., Park, S. Y., Kim, J. W., Youn, J. C., Jhoo, J. H., Lee, J. H., Kim, K. W., Woo, J. I., & Lee, D. Y. (2014). A normative study of total scores of the CERAD neuropsychological assessment battery in an educationally diverse elderly population. *International Psychogeriatrics*, *26*(11), 1897–1904. https://doi.org/10.1017/S1041610214001379

Hartley, A. A. (2001). Age differences in dual-task interference are localized to response-generation processes. *Psychology and Aging*, *16*(1), 47–54. https://doi.org/10.1037/0882-7974.16.1.47

Hartley, A. A., Little, D. M., Dean, D., Eberle, C., Garcia, M., Liegler, K., Metzger, T., Robinson, T., Teal, J., Tranh, N., & Wong, S. (1999). Age-Related Differences and Similarities in Dual-Task Interference. *Journal of Experimental Psychology: General*, *128*(4), 416–449.

Hartley, A. A., Seaman, B., & Maquestiaux, F. (2015). Ideomotor-compatible tasks partially escape dual-task interference in both young and elderly adults. *Psychology and Aging*, *30*(1), 36–45. https://doi.org/10.1037/pag0000015

Hasher, L., Lustig, C., & Zacks, R. (2008). Inhibitory Mechanisms and the Control of Attention. In *Variation in Working Memory* (Vol. 15, Issue 1, pp. 583–605). https://doi.org/10.1093/acprof:oso/9780195168648.003.0009

Hasher, L., & Zacks, R. (1988). Working Memory, Comprehension, and Aging: A Review and a New View. In *The Psychology of Learning* (pp. 193–225). Academic Press.

Hashimoto, R., Meguro, K., Lee, E., Kasai, M., Ishii, H., & Yamaguchi, S. (2006). Effect of age and education on the Trail Making Test and determination of normative data for Japanese elderly people: The Tajiri Project. *Psychiatry and Clinical Neurosciences*, *60*(4), 422–428. https://doi.org/10.1111/j.1440-1819.2006.01526.x

Hein, G., & Schubert, T. (2004). Aging and input processing in dual-task situations. *Psychology and Aging*, *19*(3), 416–432. https://doi.org/10.1037/0882-7974.19.3.416

Hester, R. L., Kinsella, G. J., Ong, B., & McGregor, J. (2005). Demographic influences on baseline and derived scores from the trail making test in healthy older Australian adults. *Clinical Neuropsychologist*, *19*(1), 45–54. https://doi.org/10.1080/13854040490524137

Hirsch, P., Schwarzkopp, T., Declerck, M., Reese, S., & Koch, I. (2016). Age-related differences in task switching and task preparation: Exploring the role of task-set competition. *Acta Psychologica*, *170*, 66–73. https://doi.org/10.1016/j.actpsy.2016.06.008

Houx, P., Jolles, J., & Vreeling, F. (1993). Stroop interference: aging effects assessed with the Stroop Color-Word Test. *Experimental Aging Research*, *19*(3), 209–224. https://doi.org/10.1080/03610739308253934

Huff, M. J., Balota, D. A., Minear, M., Aschenbrenner, A. J., & Duchek, J. M. (2015). Dissociative global and local task-switching costs across younger adults, middle-aged adults, older adults, and very mild Alzheimer’s disease individuals. *Psychology and Aging*, *30*(4), 727–739. https://doi.org/10.1037/pag0000057

Kennedy, K. (1981). Age Effects on Trail Making Test Performance. *Perceptual and Motor Skills*, *52*(2), 671–675.

Kirchner, W. K. (1958). Age differences in short-term retention of rapidly changing information. *Journal of Experimental Psychology*, *55*(4), 352.

Koch, I., Gade, M., Schuch, S., & Philipp, A. M. (2010). The role of inhibition in task switching: A review. *Psychonomic Bulletin and Review*, *17*(1), 1–14. https://doi.org/10.3758/PBR.17.1.1

Kramer, A. F., Hahn, S., & Gopher, D. (1999). Task coordination and aging: Explorations of executive control processes in the task switching paradigm. *Acta Psychologica*, *101*(2–3), 339–378. https://doi.org/10.1016/s0001-6918(99)00011-6

Kray, J., Li, K. Z. H., & Lindenberger, U. (2002). Age-related changes in task-switching components: The role of task uncertainty. *Brain and Cognition*, *49*(3), 363–381. https://doi.org/10.1006/brcg.2001.1505

Kray, J., & Lindenberger, U. (2000). Adult age differences in task switching. *Psychology and Aging*, *15*(1), 126–147. https://doi.org/10.1037/0882-7974.15.1.126

Li, K. Z. H., Vadaga, K. K., Bruce, H., & Lai, L. (2019). Executive Function Development in Aging. *Executive Function*, *July 2017*, 59–72. https://doi.org/10.4324/9781315160719-5

Linares, R., Bajo, M. T., & Pelegrina, S. (2016). Age-related differences in working memory updating components. *Journal of Experimental Child Psychology*, *147*, 39–52. https://doi.org/10.1016/j.jecp.2016.02.009

Lustig, C., Hasher, L., & Zacks, R. (2007). Inhibitory deficit theory: Recent developments in a “new view.” *Inhibition in Cognition.*, 145–162. https://doi.org/10.1037/11587-008

Maquestiaux, F. (2016). Qualitative attentional changes with age in doing two tasks at once. *Psychonomic Bulletin and Review*, *23*(1), 54–61. https://doi.org/10.3758/s13423-015-0881-9

Maquestiaux, F., Laguë-Beauvais, M., Ruthruff, E., & Bherer, L. (2008). Bypassing the central bottleneck after single-task practice in the psychological refractory period paradigm: Evidence for task automatization and greedy resource recruitment. *Memory and Cognition*, *36*(7), 1262–1282. https://doi.org/10.3758/MC.36.7.1262

Maquestiaux, F., Laguë-Beauvais, M., Ruthruff, E., Hartley, A., & Bherer, L. (2010). Learning to Bypass the Central Bottleneck: Declining Automaticity With Advancing Age. *Psychology and Aging*, *25*(1), 177–192. https://doi.org/10.1037/a0017122

Martin, A. K., Barker, M. S., Gibson, E. C., & Robinson, G. A. (2019). Response initiation and inhibition and the relationship with fluid intelligence across the adult lifespan. *Archives of Clinical Neuropsychology*, *00*, 1–12. https://doi.org/10.1093/arclin/acz044

Mayas, J., Fuentes, L. J., & Ballesteros, S. (2012). Stroop interference and negative priming (NP) suppression in normal aging. *Archives of Gerontology and Geriatrics*, *54*(2), 333–338. https://doi.org/10.1016/j.archger.2010.12.012

Mayr, U. (2001). Age Differences in the Selection of Mental Sets: The Role of Inhibition, Stimulus Ambiguity, and Response-Set Overlap. *Psychology and Aging*, *16*(1), 96–109.

McCabe, D. P., Robertson, C. L., & Smith, A. D. (2005). Age differences in stroop interference in working memory. *Journal of Clinical and Experimental Neuropsychology*, *27*(5), 633–644. https://doi.org/10.1080/13803390490919218

Meiran, N., Gotler, A., & Perlman, A. (2001). Old age is associated with a pattern of relatively intact and relatively impaired task-set switching abilities. *Journals of Gerontology - Series B Psychological Sciences and Social Sciences*, *56*(2), 88–102. https://doi.org/10.1093/geronb/56.2.P88

Monsell, S. (2003). Task switching. *Trends in Cognitive Sciences*, *7*(3), 134–140. https://doi.org/10.1016/S1364-6613(03)00028-7

Moretti, L., Semenza, C., & Vallesi, A. (2018). General slowing and education mediate task switching performance across the life-span. *Frontiers in Psychology*, *9*(MAY), 1–12. https://doi.org/10.3389/fpsyg.2018.00630

Morrone, I., Declercq, C., Novella, J. L., & Besche, C. (2010). Aging and inhibition processes: The case of metaphor treatment. *Psychology and Aging*, *25*(3), 697–701. https://doi.org/10.1037/a0019578

Nigg, J. T. (2000). On Inhibition/Disinhibition in Developmental Psychopathology: Views from Cognitive and Personality Psychology and a Working Inhibition Taxonomy. *Psychological Bulletin*, *126*(2), 220–246. https://doi.org/10.1037/0033-2909.126.2.220

Ostrosky‐Solís, F., & Lozano, A. (2006). Digit span: Effect of education and culture. *International Journal of Psychology*, *41*(5), .333-341.

Pashler, H. (1984). Processing stages in overlapping tasks: Evidence for a central bottleneck. *Journal of Experimental Psychology: Human Perception and Performance*, *10*(3), 358–377. https://doi.org/10.1037/0096-1523.10.3.358

Persad, C. C., Abeles, N., Zacks, R. T., & Denburg, N. L. (2002). Inhibitory changes after age 60 and their relationship to measures of attention and memory. *Journals of Gerontology - Series B Psychological Sciences and Social Sciences*, *57*(3), P223–P232. https://doi.org/10.1093/geronb/57.3.P223

Pettigrew, C., & Martin, R. C. (2014). Cognitive declines in healthy aging: Evidence from multiple aspects of interference resolution. *Psychology and Aging*, *29*(2), 187–204. https://doi.org/10.1037/a0036085

Rasmusson, D. X., Zonderman, A. B., Kawas, C., & Resnick, S. M. (1998). Effects of age and dementia on the trail making test. *Clinical Neuropsychologist*, *12*(2), 169–178. https://doi.org/10.1076/clin.12.2.169.2005

Reimers, S., & Maylor, E. A. (2005). Task switching across the life Span: Effects of age on general and specific switch costs. *Developmental Psychology*, *41*(4), 661–671. https://doi.org/10.1037/0012-1649.41.4.661

Reitan, R. M. (1992). Trail Making Test: Manual for administration and scoring. In *Reitan Neuropsychology Laboratory*.

Rey-Mermet, A., & Gade, M. (2018). Inhibition in aging: What is preserved? What declines? A meta-analysis. *Psychonomic Bulletin and Review*, *25*(5), 1695–1716. https://doi.org/10.3758/s13423-017-1384-7

Robertson, I. H., Ward, T., Ridgeway, V., & Nimmo-Smith, I. (1994). The Test of Everyday Attention: TEA. *October*, *4*(1), 51–55. https://www.researchgate.net/publication/267552527

Rogers, R. D., & Monsell, S. (1995). Costs of a Predictable Switch Between Simple Cognitive Tasks. *Journal of Experimental Psychology: General*, *124*(2), 207–231. https://doi.org/10.1037/0096-3445.124.2.207

Rubinstein, J. S., Meyer, D. E., & Evans, J. E. (2001). Executive Control of Cognitive Processes in Task Switching. *Journal of Experimental Psychology: Human Perception and Performance*, *27*(4), 763–797. https://doi.org/10.1037//0096-1523.27.4.763

Salthouse, T. A. (1996). The processing-speed theory of adult age differences in cognition. *Psychological Review*, *103*(3), 403–428. https://doi.org/10.1037/0033-295X.103.3.403

Spieler, D. H., Balota, D. A., & Faust, M. E. (1996). Stroop Performance in Healthy Younger and Older Adults and in Individuals with Dementia of the Alzheimer’s Type. *Journal of Experimental Psychology: Human Perception and Performance*, *22*(2), 461–479. https://doi.org/10.1037/0096-1523.22.2.461

Szameitat, A. J., & Students, B. (2022). Inter-Individual Differences in Executive Functions Predict Multitasking Performance – Implications for the Central Attentional Bottleneck. *Frontiers in Psychology*, *13*(May), 1–15. https://doi.org/10.3389/fpsyg.2022.778966

Tombaugh, T. N. (2004). Trail Making Test A and B: Normative data stratified by age and education. *Archives of Clinical Neuropsychology*, *19*(2), 203–214. https://doi.org/10.1016/S0887-6177(03)00039-8

Tournier, I., Postal, V., & Mathey, S. (2014). Investigation of age-related differences in an adapted Hayling task. *Archives of Gerontology and Geriatrics*, *59*, 599–606. https://doi.org/10.1016/j.archger.2014.07.016

Troyer, A. K., Leach, L., & Strauss, E. (2006). Aging and response inhibition: Normative data for the Victoria Stroop Test. *Aging, Neuropsychology, and Cognition*, *13*(1), 20–35. https://doi.org/10.1080/138255890968187

Uttl, B., & Graf, P. (1997). Color-Word Stroop test performance across the adult life span. *Journal of Clinical and Experimental Neuropsychology*, *19*(3), 405–420. https://doi.org/10.1080/01688639708403869

Van Asselen, M., & Ridderinkhof, K. R. (2000). Shift costs of predictable and unexpected set shifting in young and older adults. In *Psychologica Belgica* (Vol. 40, Issue 4, pp. 259–273). https://doi.org/10.5334/pb.966

Vaughan, L., Basak, C., Hartman, M., & Verhaeghen, P. (2008). Aging and working memory inside and outside the focus of attention: Dissociations of availability and accessibility. *Aging, Neuropsychology, and Cognition*, *15*(6), 703–724. https://doi.org/10.1080/13825580802061645

Velichkovsky, B. B., Tatarinov, D. V., Khlebnikova, A. A., Roshchina, I. F., Selezneva, N. D., & Gavrilova, S. I. (2020). Task switching in normal aging and mild cognitive impairment: A diffusion model analysis of reaction times. *Psychology in Russia: State of the Art*, *13*(2), 109–120. https://doi.org/10.11621/pir.2020.0208

Verhaeghen, P., & Basak, C. (2005). Ageing and switching of the focus of attention in working memory: Results from a modified N-Back task. *Quarterly Journal of Experimental Psychology Section A: Human Experimental Psychology*, *58*(1), 134–154. https://doi.org/10.1080/02724980443000241

Verhaeghen, P., Steitz, D. W., Sliwinski, M. J., & Cerella, J. (2003). Aging and dual-task performance: A meta-analysis. In *Psychology and Aging* (Vol. 18, Issue 3, pp. 443–460). https://doi.org/10.1037/0882-7974.18.3.443

Wasylyshyn, C., Verhaeghen, P., & Sliwinski, M. J. (2011). Aging and Task Switching: A Meta-Analysis. *Psychology and Aging*, *26*(1), 15–20. https://doi.org/10.1037/a0020912

Wecker, N. S., Kramer, J. H., Hallam, B. J., & Delis, D. C. (2005). Mental flexibility: Age effects on switching. *Neuropsychology*, *19*(3), 345–352. https://doi.org/10.1037/0894-4105.19.3.345

Wecker, N. S., Kramer, J. H., Wisniewski, A., Delis, D. C., & Kaplan, E. (2000). Age effects on executive ability. *Neuropsychology*, *14*(3), 409–414. https://doi.org/10.1037/0894-4105.14.3.409

Welford, A. T. (1952). The psychological refractory period and the timing of high- speed performance - A review and a theory. *British Journal of Social Psycholology*, *43*, 2–19. https://doi.org/10.1111/j.2044-8295.1952.tb00322.x

Woods, D. L., Kishiyama, M. M., Yund, E. W., Herron, T. J., Edwards, B., Poliva, O., Hink, R. F., & Reed, B. (2011). Improving digit span assessment of short-term verbal memory. *Journal of Clinical and Experimental Neuropsychology*, *33*(1), 101–111. https://doi.org/10.1080/13803395.2010.493149

Woods, D. L., Wyma, J. M., Herron, T. J., & Yund, E. W. (2015). The effects of aging, malingering, and traumatic brain injury on computerized trail-making test performance. *PLoS ONE*, *10*(6), 1–30. https://doi.org/10.1371/journal.pone.0124345

Zimmermann, N., Cardoso, C. de O., Kristensen, C. H., & Fonseca, R. P. (2017). Brazilian norms and effects of age and education on the Hayling and Trail Making Tests. *Trends in Psychiatry and Psychology*, *39*(3), 188–195.

**Tables**

**Table S1. Dual-Tasking Results of the Young and Older Adult Participants.**

| Task | Young Adults | | | Older Adults | | | *t* | *df* | Young vs Old, *p*-value |
| --- | --- | --- | --- | --- | --- | --- | --- | --- | --- |
|  | N | O | Mean (SD) | N | O | Mean (SD) |  |  |  |
| *Test of Everyday Attention (TEA)^1^* | | | | | | | | | |
| Auditory ST count accuracy | 24 | 2 | N/A | 20 | 4 | 97.50% (7.69) |  |  | - |
| Auditory DT count accuracy | 24 | 2 | 90.42% (13.34) | 20 | 4 | 92.50% (11.18) | -0.55 | 42 | 0.582 |
| Auditory DT count accuracy DT cost | 24 | 2 | N/A | 20 | 4 | -5.00% (10.88) |  |  | - |
| Telephone code count ST accuracy | 24 | 2 | N/A | 20 | 4 | 71.43% (17.34) |  |  | - |
| Telephone code count DT accuracy | 24 | 2 | 79.17% (15.12) | 20 | 4 | 75.88% (15.26) | 0.72 | 42 | 0.479 |
| Telephone code count accuracy DT cost | 24 | 2 | N/A | 20 | 4 | 4.45% (20.89) |  |  | - |
| *Psychological Refractory Period paradigm (PRP)^2^* | | | | | | | | | |
| Auditory ST RT | 24 | 0 | 616.97 ms (199.59) | 22 | 3 | 657.28 ms (152.67) | -0.76 | 44 | 0.449 |
| Auditory RT1 DT (SOA 0 ms) | 24 | 0 | 1079.62 ms (365.27) | 22 | 3 | 1097.13 ms (287.27) | -0.18 | 44 | 0.858 |
| Auditory RT1 (SOA 0 ms) DT (DT – ST) cost | 24 | 0 | 462.65 ms (323.27) | 22 | 3 | 439.85 ms (245.96) | 0.27 | 44 | 0.790 |
| Visual ST RT | 24 | 0 | 503.54 ms (109.17) | 22 | 3 | 553.77 ms (59.37) | -1.93 | 44 | 0.062 |
| Visual RT2 DT (SOA 0 ms) | 24 | 0 | 1312.99 ms (418.22) | 22 | 3 | 1459.13 ms (311.22) | -1.33 | 44 | 0.189 |
| Visual RT2 (SOA 0 ms) DT (DT – ST) cost | 24 | 0 | 809.45 ms (381.28) | 22 | 3 | 905.37 ms (292.60) | -0.95 | 44 | 0.347 |
| Visual RT2 DT (SOA 1000 ms) | 24 | 0 | 743.43 ms (360.35) | 22 | 3 | 726.62 ms (209.67) | 0.19 | 44 | 0.849 |
| PRP effect (DT SOA 0 ms – DT SOA 1000 ms), visual task, RT2 | 24 | 0 | 569.56 ms (254.83) | 22 | 3 | 732.51 ms (250.94) | -2.18 | 44 | **0.034** |
| Auditory ST error-rate | 24 | 0 | 7.33% (7.91) | 22 | 3 | 3.00% (5.94) | 2.09 | 44 | 0.043 |
| Auditory R1 DT (SOA 0 ms) | 24 | 0 | 10.75% (9.81) | 22 | 3 | 3.91% (6.66) | 2.74 | 44 | **0.009** |
| Auditory R1 (SOA 0 ms) DT (DT – ST) cost | 24 | 0 | 3.42% (6.92) | 22 | 3 | 0.09% (3.53) | 1.53 | 44 | 0.134 |
| Visual R2 ST | 24 | 0 | 3.42% (4.55) | 22 | 3 | 0.64% (1.14) | 2.79 | 44 | **0.008** |
| Visual R2 DT (SOA 0 ms) | 24 | 0 | 7.75% (7.13) | 22 | 3 | 2.36% (2.36) | 3.37 | 44 | **0.002** |
| Visual R2 (SOA 0 ms) DT (DT – ST) cost | 24 | 0 | 4.33% (5.71) | 22 | 3 | 1.73% (2.41) | 1.98 | 44 | 0.054 |
| Visual R2 DT (SOA 1000 ms) | 24 | 0 | 9.08% (11.25) | 22 | 3 | 1.27% (2.10) | 3.20 | 44 | **0.003** |
| PRP effect (DT SOA 0 ms – DT SOA 1000 ms), visual task, R2 | 24 | 0 | -1.33% (9.60) | 22 | 3 | 1.09% (2.20) | -1.16 | 44 | 0.254 |

N – number of participants (after removal of outliers); O – number of all outliers (i.e., N + O = original sample size); ST – Single task; DT – Dual-task; RT – Response time; R – error-rate; SOA – Stimulus Onset Asynchrony; N/A – The young adults did not complete the TEA single-tasks. Please note RT1 corresponds with R1, and RT2 with R2. ^1^ Please note in addition to performance outliers, participants who produced an error-rate of 60% or higher in any of the task conditions were removed from analysis. Hence, the data of two young adults, and one older adult were not analyzed. ^2^ In addition, participants who produced an error-rate of 50% or greater were excluded from analysis hence the data from two young adults were not analyzed. Bold *p*-values indicate significant effects (*p* < 0.05). (Bonferroni alpha *p* < 0.003).

**Table S2. Inhibition Results of the Young and Older Adult Participants.**

| Task | Young Adults | | | Older Adults | | | *t* | *df* | Young vs Old, *p*-value |
| --- | --- | --- | --- | --- | --- | --- | --- | --- | --- |
|  | N | O | Mean (SD) | N | O | Mean (SD) |  |  |  |
| *Hayling sentence completion test (HSCT)^1^* | | | | | | | | | |
| Part 1 RT | 26 | 1 | 22.27 s (8.22) | 23 | 1 | 19.00 s (3.37) | 1.78 | 47 | 0.082 |
| Part 2 RT | 26 | 1 | 29.04 s (18.53) | 23 | 1 | 47.30 s (21.79) | -3.17 | 47 | **0.003** |
| Inhibition RT cost | 26 | 1 | 6.77 s (17.80) | 23 | 1 | 28.30 s (21.10) | -3.88 | 47 | **< 0.001** |
| Part 1 completion score | 26 | 1 | 4.03 (0.82) | 23 | 1 | 4.39 (0.66) | -1.64 | 47 | 0.107 |
| Part 2 completion score | 26 | 1 | 5.77 (0.65) | 23 | 1 | 5.22 (0.90) | 2.47 | 47 | **0.017** |
| Part 2, error score | 26 | 1 | 5.96 (1.97) | 23 | 1 | 5.48 (2.04) | 0.84 | 47 | 0.404 |
| Overall test score | 26 | 1 | 5.08 (1.32) | 23 | 1 | 4.60 (1.53) | 1.15 | 47 | 0.257 |
| *Stroop task* | | | | | | | | | |
| Word (no. out of 100) | 26 | 0 | 93.19 (10.00) | 25 | 0 | 90.24 (13.74) | 0.88 | 49 | 0.383 |
| Colour (no. out of 100) | 26 | 0 | 71.54 (13.97) | 25 | 0 | 65.44 (11.29) | 1.71 | 49 | 0.093 |
| Colour-Word (no. out of 100) | 26 | 0 | 47.04 (10.92) | 25 | 0 | 32.28 (10.01) | 5.03 | 49 | **< 0.001** |
| Colour-Word’ (no. out of 100) | 26 | 0 | 39.69 (5.39) | 25 | 0 | 37.80 (5.69) | 1.22 | 49 | 0.228 |

N – number of participants (after removal of outliers); O – number of all outliers (i.e., N + O = original sample size); RT – Response time. ^1^ In addition, the data from one young and one older adult participant were removed from the analysis due to audio recording issues. Bold *p*-values indicate significant effects (*p* < 0.05). (Bonferroni alpha *p* < 0.005).

**Table S3. Shifting Results of the Young and Older Adult Participants.**

| Task | Young Adults | | | Older Adults | | | *t* | *df* | Young vs Old, *p*-value |
| --- | --- | --- | --- | --- | --- | --- | --- | --- | --- |
|  | N | O | Mean (SD) | N | O | Mean (SD) |  |  |  |
| *Task switching task^1^* | | | | | | | | | |
| Local shift – RT repetition | 26 | 2 | 1253.00 ms (350.88) | 21 | 2 | 1475.14 ms (336.95) | -2.20 | 45 | **0.033** |
| Local shift – RT shift | 26 | 2 | 1328.95 ms (340.29) | 21 | 2 | 1681.33 ms (507.58) | -2.84 | 45 | **0.007** |
| Local shift RT cost | 26 | 2 | 75.96 ms (84.29) | 21 | 2 | 206.20 ms (239.41) | -2.58 | 45 | **0.013** |
| Mixed block – RT repetition | 26 | 2 | 966.32 ms (179.70) | 21 | 2 | 1071.00 ms (133.12) | -2.22 | 45 | **0.031** |
| Mixing block – RT shift | 26 | 2 | 1253.00 ms (350.88) | 21 | 2 | 1475.14 ms (336.95) | -2.20 | 45 | **0.033** |
| RT Mixing-cost | 26 | 2 | 286.67 ms (222.77) | 21 | 2 | 404.14 ms (314.73) | -1.50 | 45 | 0.142 |
| Global shift – RT repetition | 26 | 2 | 966.32 ms (179.70) | 21 | 2 | 1071.00 ms (133.12) | -2.22 | 45 | **0.031** |
| Global shift – RT shift | 26 | 2 | 1329.38 ms (345.20) | 21 | 2 | 1690.79 ms (496.87) | -2.94 | 45 | **0.005** |
| Global shift RT cost | 26 | 2 | 363.06 ms (211.52) | 21 | 2 | 619.79 ms (457.52) | -2.55 | 45 | **0.014** |
| Local shift – error-rate repetition | 26 | 2 | 3.69% (4.22) | 21 | 2 | 6.14% (9.12) | -1.22 | 45 | 0.229 |
| Local shift – error-rate shift | 26 | 2 | 8.42% (7.72) | 21 | 2 | 9.10% (10.39) | -0.26 | 45 | 0.800 |
| Error-rate Local shift cost | 26 | 2 | 4.77% (5.92) | 21 | 2 | 3.10% (4.58) | 1.06 | 45 | 0.293 |
| Mixed block – error-rate repetition | 26 | 2 | 2.85% (2.74) | 21 | 2 | 1.43% (1.33) | 2.17 | 45 | **0.035** |
| Mixed block – error-rate shift | 26 | 2 | 3.69% (4.22) | 21 | 2 | 6.14% (9.12) | -1.22 | 45 | 0.229 |
| Error-rate Mixing-cost | 26 | 2 | 0.88% (3.71) | 21 | 2 | 4.81% (8.44) | -2.13 | 45 | **0.038** |
| Global shift – error-rate repetition | 26 | 2 | 2.85% (2.74) | 21 | 2 | 1.43% (1.33) | 2.17 | 45 | **0.035** |
| Global shift – error-rate shift | 26 | 2 | 8.46% (7.65) | 21 | 2 | 8.86% (9.97) | -0.15 | 45 | 0.878 |
| Error-rate Global shift cost | 26 | 2 | 5.54% (5.59) | 21 | 2 | 7.52% (9.14) | -0.92 | 45 | 0.364 |
| *Trail making test (TMT)^2^* | | | | | | | | | |
| Part A RT | 24 | 2 | 32.29 s (10.80) | 23 | 0 | 32.70 s (10.34) | -0.13 | 45 | 0.896 |
| Part B RT | 19 | 0 | 64.11 s (19.54) | 19 | 0 | 57.53 s (15.12) | 1.16 | 36 | 0.253 |
| RT Shifting cost | 19 | 0 | 31.00 s (17.68) | 19 | 0 | 25.89 s (11.99) | 1.04 | 36 | 0.305 |
| Part A error-rate | 24 | 2 | 2.08% (10.21) | 23 | 0 | 6.52% (17.22) | -1.08 | 45 | 0.286 |
| Part B error-rate | 19 | 0 | 15.79% (33.55) | 19 | 0 | 26.32% (45.21) | -0.82 | 36 | 0.421 |
| Error-rate shifting cost | 19 | 0 | 13.16% (36.67) | 19 | 0 | 18.42% (47.76) | -0.38 | 45 | 0.705 |

N – number of participants (after removal of outliers); O – number of all outliers (i.e., N + O = original sample size); RT – Response time. ^1^ In addition, the data from two older adults could not be analyzed due to data recovery issues. ^2^ In addition, the data from one young and two older adults from part A, plus 5 young and 4 older adults from part B were not assessed as these participants produced three or more errors in either task part. Bold *p*-values indicate significant effects (*p* < 0.05). (Bonferroni alpha *p* < 0.002).

**Table S4. N-Back task results of the Young and Older Adult Participants.**

| N-back condition | Young Adults | | | Older Adults | | | *t* | *df* | Young vs Old, *p*-value |
| --- | --- | --- | --- | --- | --- | --- | --- | --- | --- |
|  | N | O | Mean (SD) | N | O | Mean (SD) |  |  |  |
| 0-back, RT | 26 | 0 | 536.86 ms (89.89) | 24 | 1 | 785.18 ms (159.74) | -6.84 | 48 | **< 0.001** |
| 1-, RT | 26 | 0 | 525.91 ms (153.08) | 24 | 1 | 764.81 ms (216.70) | -4.53 | 48 | **< 0.001** |
| 2-, RT | 26 | 0 | 573.06 ms (186.48) | 24 | 1 | 825.30 ms (242.33) | -4.14 | 48 | **< 0.001** |
| 3-, RT | 26 | 0 | 562.74 ms (189.19) | 23 | 1 | 798.87 ms (247.72) | -3.77 | 47 | **< 0.001** |
| 0-back, error-rate | 26 | 0 | 2.40% (2.32) | 24 | 1 | 3.61% (3.72) | -1.39 | 48 | 0.171 |
| 1-, error-rate | 26 | 0 | 14.90% (8.13) | 24 | 1 | 31.15% (22.38) | -3.46 | 48 | **0.001** |
| 2-, error-rate | 26 | 0 | 40.05% (18.65) | 24 | 1 | 60.28% (20.96) | -3.61 | 48 | **0.001** |
| 3-, error-rate | 26 | 0 | 52.84% (14.81) | 23 | 1 | 70.38% (14.47) | -4.18 | 47 | **< 0.001** |

N – number of participants (after removal of outliers); O – number of all outliers (i.e., N + O = original sample size); RT – Response time. Bold *p*-values indicate significant effects (*p* < 0.05). (Bonferroni alpha *p* < 0.005).
